# Informatics for RNA Sequencing: A Web Resource for Analysis on the Cloud

**DOI:** 10.1371/journal.pcbi.1004393

**Published:** 2015-08-06

**Authors:** Malachi Griffith, Jason R. Walker, Nicholas C. Spies, Benjamin J. Ainscough, Obi L. Griffith

**Affiliations:** 1 McDonnell Genome Institute, Washington University School of Medicine, St. Louis, Missouri, United States of America; 2 Siteman Cancer Center, Washington University School of Medicine, St. Louis, Missouri, United States of America; 3 Department of Genetics, Washington University School of Medicine, St. Louis, Missouri, United States of America; 4 Department of Medicine, Washington University School of Medicine, St. Louis, Missouri, United States of America; Ontario Institute for Cancer Research, CANADA

## Abstract

Massively parallel RNA sequencing (RNA-seq) has rapidly become the assay of choice for interrogating RNA transcript abundance and diversity. This article provides a detailed introduction to fundamental RNA-seq molecular biology and informatics concepts. We make available open-access RNA-seq tutorials that cover cloud computing, tool installation, relevant file formats, reference genomes, transcriptome annotations, quality-control strategies, expression, differential expression, and alternative splicing analysis methods. These tutorials and additional training resources are accompanied by complete analysis pipelines and test datasets made available without encumbrance at www.rnaseq.wiki.

*This is part of the PLOS Computational Biology Education collection.*

## Introduction to RNA Sequencing

**Fig 1:**
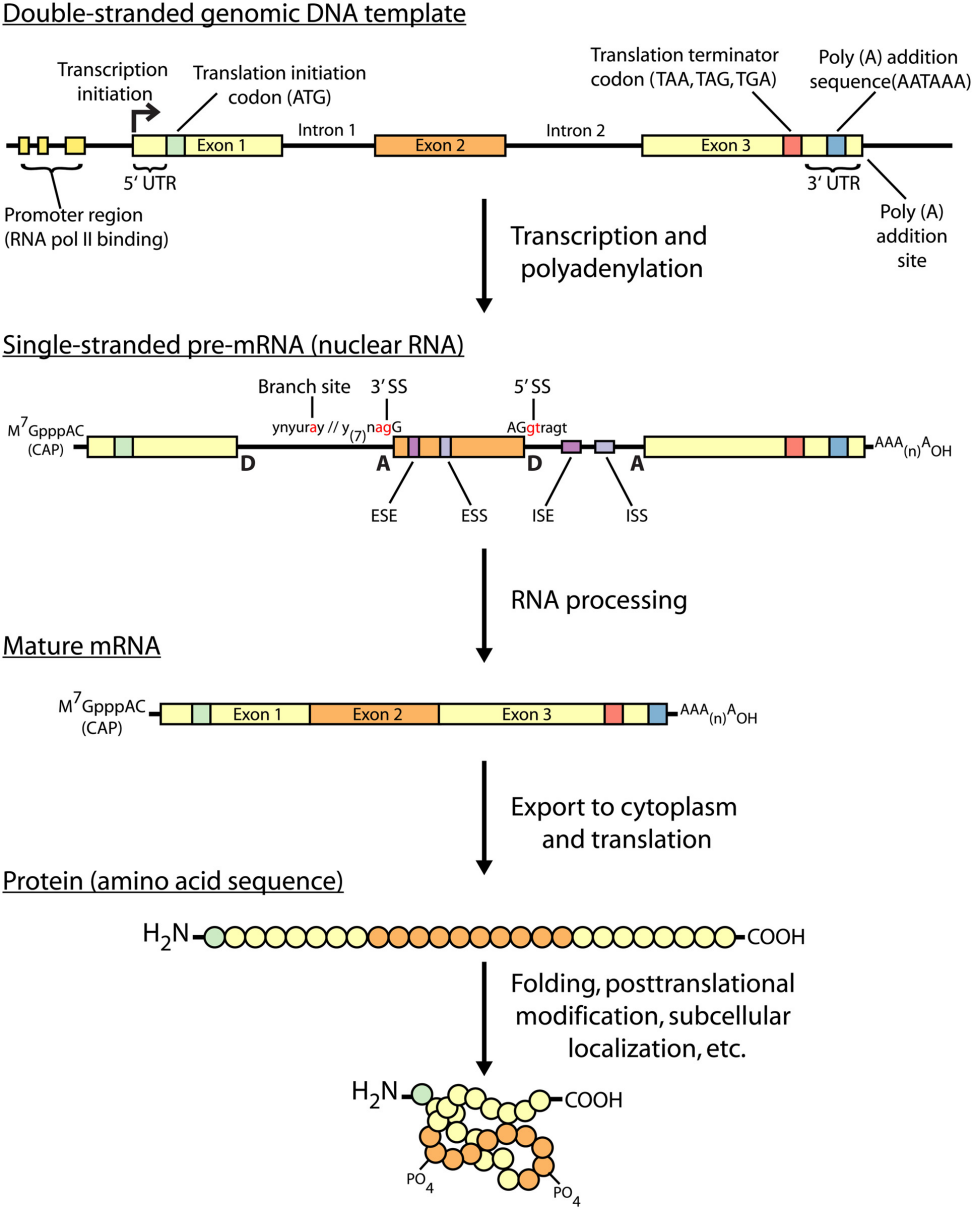
An overview of the central dogma of molecular biology. The flow of genetic information from double-stranded genomic DNA template to post-translationally modified proteins is depicted with molecular features critical to each stage enumerated. RNA-seq typically targets the mature mRNA molecules. Abbreviations: donor splice site (D); acceptor splice site (A); polyadenylation (poly (A)); untranslated region (UTR); splice site (SS); exonic splicing enhancer (ESE), exonic splicing silencer (ESS), intronic splicing enhancer (ISE); intronic splicing silencer (ISS).

Gene expression is a widely studied process and a major area of focus for functional genomics [1]. Gene expression is concerned with the flow of genetic information from the genomic DNA template to functional protein products (Fig 1). Massively parallel RNA sequencing (RNA-seq) has become a standard gene expression assay, particularly for interrogating relative transcript abundance and diversity. Several studies have confirmed that its measurement accuracy rivals that of other well-established methods such as microarrays and quantitative polymerase chain reaction (qPCR) [2–4]. It has been reported that 85% of novel splicing events and 88% of differentially expressed exons predicted by RNA-seq are validated by “gold-standard” approaches such as reverse transcription polymerase chain reaction (RT-PCR) and qPCR [3].

**Fig 2:**
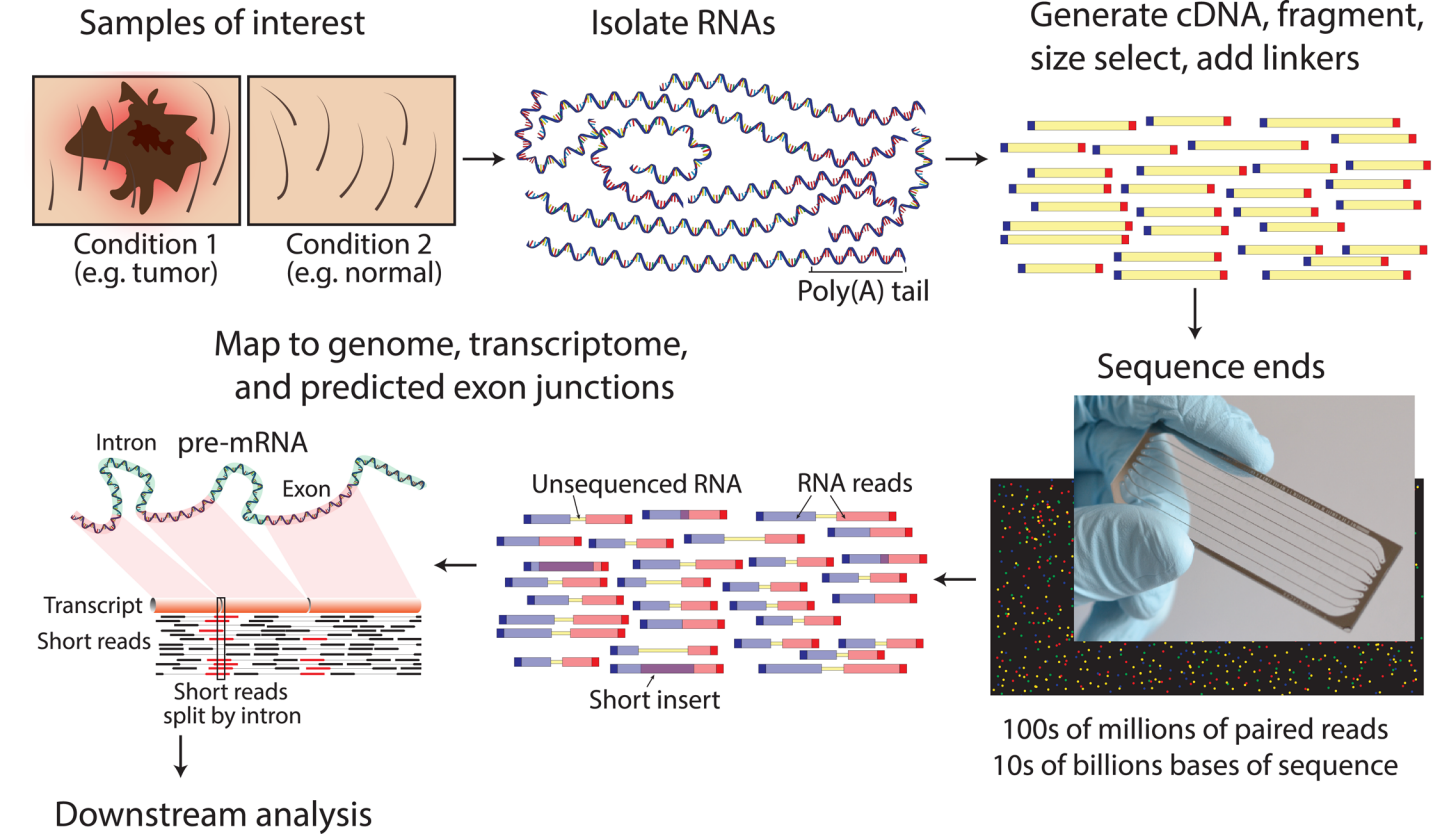
RNA-seq data generation. A typical RNA-seq experimental workflow involves the isolation of RNA from samples of interest, generation of sequencing libraries, use of a high-throughput sequencer to produce hundreds of millions of short paired-end reads, alignment of reads against a reference genome or transcriptome, and downstream analysis for expression estimation, differential expression, transcript isoform discovery, and other applications. Refer to S1 Table, S3 Table, and S7 Table for more details on the concepts depicted in this figure.

The RNA-seq method typically consists of identification of suitable biological samples (and replicates), isolation of total RNA, enrichment of nonribosomal RNAs, conversion of RNA to cDNA, construction of a fragment library, sequencing on a high-throughput sequencing platform, generation of single or paired-end reads of 30–300 base pairs in length, alignment or assembly of these reads, and downstream analysis (Fig 2) [5,6]. There are several downstream analysis goals for which RNA-seq is suitable. These include transcript discovery [7–11], genome annotation [8,12,13], studying the mechanisms of gene regulation [14], differential gene expression analysis [15–17], alternative expression analysis [3], allele-specific expression analysis [18,19], detection of RNA editing [20–22], viral detection [23–25], gene fusion detection [26–30], and other types of variant detection [31–33]. S1 Table provides a more detailed summary of RNA-seq analysis applications, and S2 Table lists tools relevant to each (additional citations for supplementary tables are provided in S1 Text). In addition to these specific applications, RNA-seq has enabled important discoveries in multiple research fields. These discoveries include fusion discoveries in cancer [34–36], a greater understanding of the prevalence, mechanisms, and regulation of alternative splicing [37–39], improved understanding of the prevalence and functional significance of noncoding RNA genes [40,41], an increased (but controversial) estimation of the prevalence of RNA editing [21], and much more. RNA-seq is also being actively transitioned to clinical applications in many human diseases [42,43]. While RNA-seq is a powerful approach for many biological questions [44], the well-known limitations and caveats of previous RNA expression assays such as microarrays are still applicable [2]. These include the limitations that an RNA-seq experiment represents only a single snapshot of the steady-state expression output of a population of cells [45] and that RNA expression does not always correlate with protein expression, as well as other limitations [46,47].

In this educational piece, we explore molecular biology concepts of RNA-seq that influence RNA-seq analysis workflows and data interpretation. We also provide a detailed introduction to fundamental RNA-seq informatics concepts and common analysis questions. These concepts are covered here, in the Supplementary Materials and lectures made available at www.rnaseq.wiki, and the videos of these lectures made available at http://bioinformatics.ca/workshops/. Finally, we make available open-access tutorials that cover cloud computing for RNA-seq analysis, tool installation, relevant file formats, reference genomes, transcriptome annotations, quality control, and complete pipelines for expression, differential expression, and alternative splicing analysis (Supplementary Tutorials online at www.rnaseq.wiki). The tutorials represent an example RNA-seq workflow based on the “tuxedo” suite [15] and other commonly used tools. These representative tools were selected from many possible alternatives to introduce the fundamental concepts of each analysis step (S2 Table). The tutorials are designed to work in Mac OS, Linux, and Amazon Web Services (AWS) Elastic Compute (EC2) environments and are accompanied by test datasets made available for educational purposes (www.rnaseq.wiki).

## RNA Isolation, Library Preparation, and Sequencing Strategy

**Fig 3:**
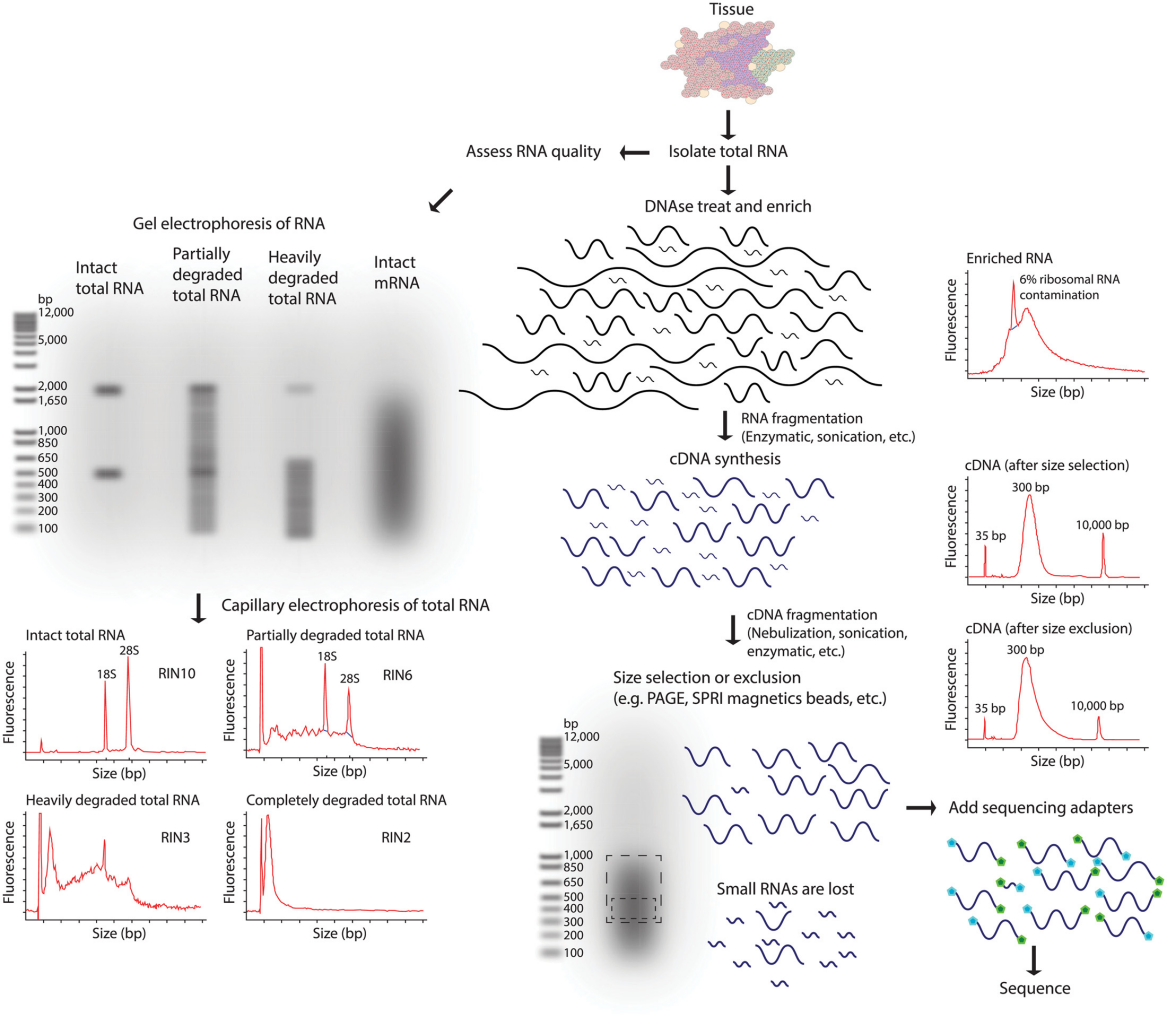
RNA-seq library fragmentation and size selection strategies that influence interpretation and analysis. RNA-seq library construction may involve both fragmentation and size selection. These procedures may be modified according to the integrity and amount of starting total RNA. The distributions of RNA molecule sizes are depicted for input total RNA and at various stages during the process of RNA/cDNA fragmentation and size selection. Commonly used methods for fragmentation and size selection are depicted along with the expected output of a quality-control assay at each stage (in the form of a capillary electrophoresis trace). Note that in the final library, it is typical that the majority of RNAs below a certain size (typically <150–200 bp) are underrepresented. Refer to S3 Table and S7 Table for more details on many of the concepts depicted in this figure.

**Fig 4:**
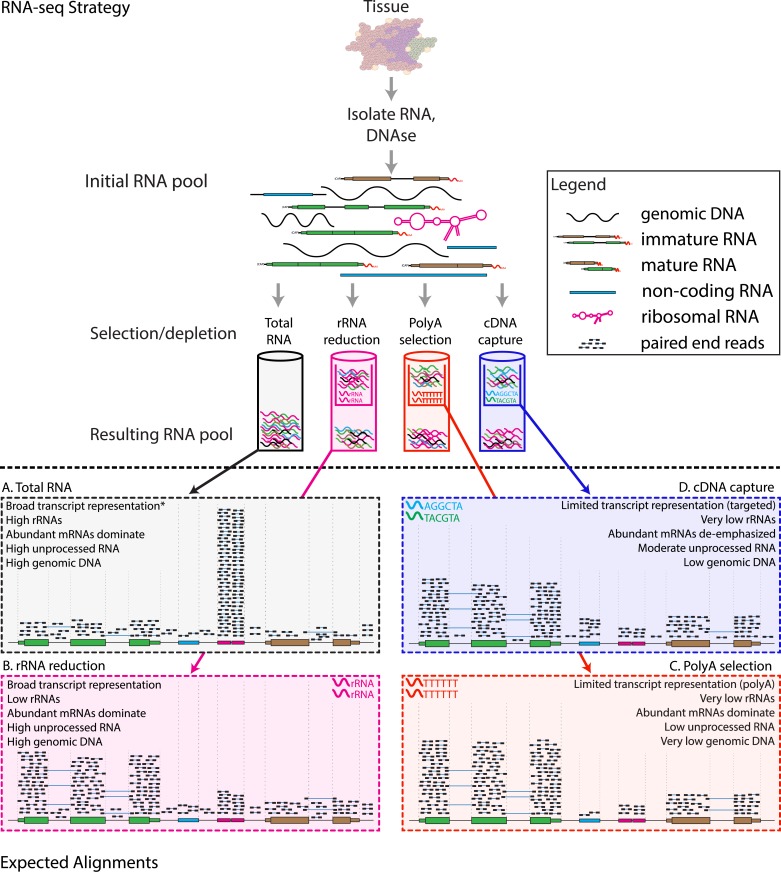
RNA-seq library enrichment strategies that influence interpretation and analysis. RNA-seq library construction protocols differ widely, and these differences have significant consequences for data interpretation and analysis. The figure above illustrates representative alignment results for either total RNA or one of three commonly used enrichment strategies at a hypothetical genomic locus with very highly expressed ribosomal RNA (pink), highly expressed protein coding (green), lowly expressed protein coding (brown) and lowly expressed noncoding RNA (blue) genes. (A) If total RNA is sequenced without enrichment, the vast majority of reads correspond to a small number of very highly expressed RNA species such as ribosomal RNAs (rRNAs). In humans, ~95%–98% of all RNA molecules may be rRNAs. A significant amount of genomic DNA (gDNA) and unprocessed heteronuclear RNA (hnRNA, also known as pre-mRNA) contamination may also remain after typical RNA isolation procedures. As a result, most reads will align to intronic, intergenic, and especially to ribosomal gene regions. Since analysis of these molecules is rarely the target of RNA-seq, various enrichment strategies are commonly employed. The amount of gDNA contamination in total RNA can be reduced, but not entirely eliminated, by use of a deoxyribonuclease (DNase) treatment. The amount of unprocessed RNA can be reduced, but not entirely eliminated, by employing an RNA isolation method that attempts to keep nuclei intact and removing these to enrich for mature mRNAs present in the cytoplasmic compartment. Additional strategies are discussed in S3 Table. * When sequencing total RNA, a complete representation of the transcriptome is theoretically present, but in practical terms, insufficient sequence reads are obtained to sufficiently sample all transcripts of all types, and some enrichment strategy is required to reduce extremely abundant rRNA species. (B) Selective rRNA reduction kits use oligonucleotides complementary to ribosomal sequences to specifically reduce the abundance of rRNAs while maintaining a broad representation of transcript species. Since the oligonucleotide probes used in these kits are only designed to bind to and deplete rRNA sequences, a significant amount of unprocessed RNA and gDNA contamination may remain. (C) Poly(A) selection and (D) cDNA capture methods specifically enrich for (primarily) mature polyadenylated RNA species or specific targets (e.g., all known transcript exons), respectively. Since poly(A) selection specifically targets RNAs that have been polyadenylated—a modification that happens at the end of the transcription process—poly(A) selection results in an enrichment for mature, completely processed RNAs. Poly(A) selection and cDNA capture methods sacrifice some transcriptome representation for increased signal to noise for transcripts of greater interest. Poly(A) methods will fail to represent most noncoding and other nonpolyadenylated RNAs. Capture methods on the other hand will under-represent any loci not specifically included in the capture design. For example, in this case the brown gene was not included in the design, and therefore, expression of this gene would be underestimated. Each of the methods depicted here has advantages and disadvantages (S3 Table and S7 Table). Furthermore, the relative amounts of each class of RNA depicted in each panel are hypothetical examples meant to demonstrate the goals and principles of each enrichment strategy and should not be interpreted quantitatively. Refer to S4 Table for additional information on the effect of each enrichment strategy.

**Fig 5:**
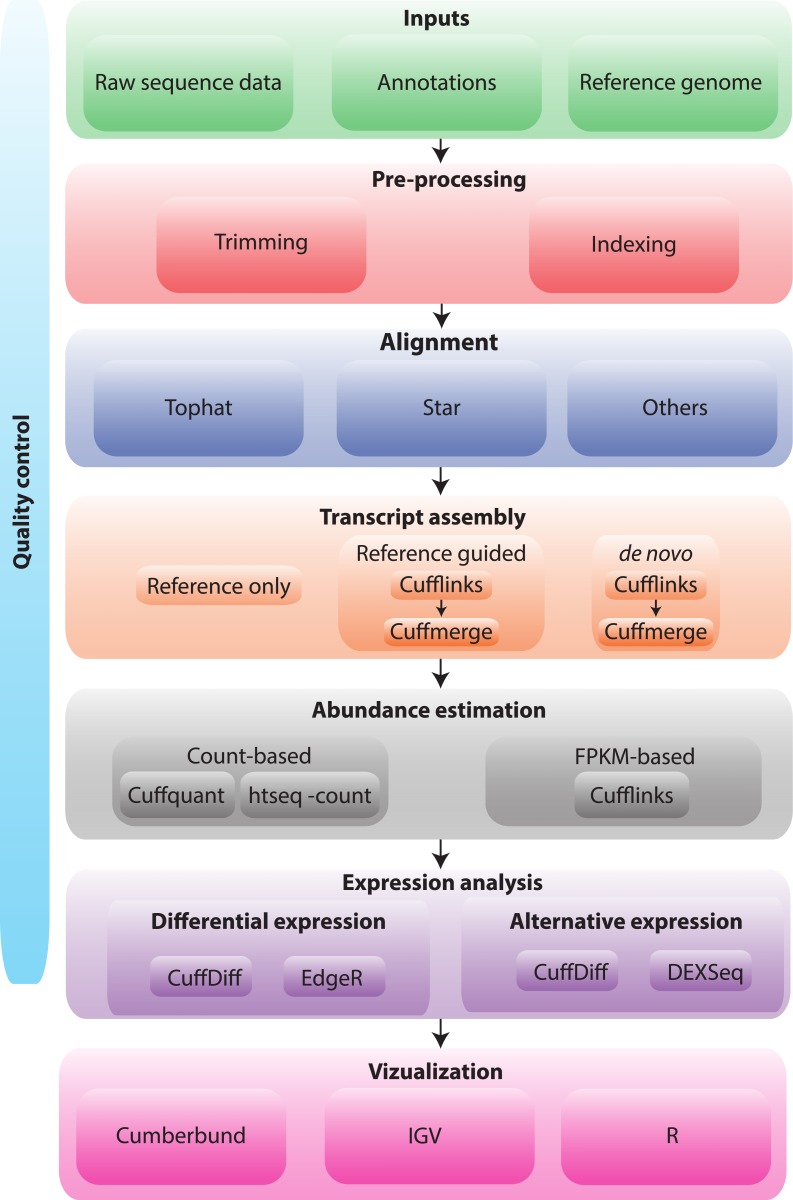
RNA-seq analysis flow chart. An example RNA-seq analysis workflow is depicted for a typical gene expression and differential expression analysis. Such workflows have several common themes across different tool sets and RNA-seq analysis goals. RNA-seq analysis typically relies on inputs such as reference genome sequences, gene annotations, and raw sequence data. Working with these inputs requires familiarity with several standardized file formats such as FASTA (.fa), FASTQ, and gene transfer format (GTF). Typical RNA-seq analysis workflows start with raw data quality control (QC), then perform read trimming, alignment or assembly of reads, apply customized algorithms for a particular analysis goal (e.g., Cufflinks and Cuffdiff for gene expression analysis), and end with summarization and visualization of the results. For each step, alternative and representative tools and strategies are shown. There are many others. Each of the workflow steps depicted here and additional analysis vignettes are implemented in the Supplementary Tutorials accompanying this work and available online at www.rnaseq.wiki. Refer to S1–S3 Tables and S7 Table for more details on many of the concepts depicted in this figure as well as alternative tools for each step.

The experimental design parameters of RNA-seq remain an area of development and may have significant impacts on analysis strategy (Fig 3 and S3 Table) [48]. These parameters include whether to perform poly(A) enrichment of total RNA or selective ribosomal RNA reduction strategies (Fig 4 and S4 Table), how to perform size selection, the use of linear amplification to rescue samples with limited RNA available [49], the use of stranded or unstranded library construction methods (Fig 5 and S5 Table), and the use of cDNA normalization techniques [50–52]. Similarly, the choice of sequencing platform (e.g., Illumina, Ion Torrent, etc.), instrument (e.g., Ion Personal Genome Machine [PGM], MiSeq, HiSeq, etc.), length of reads generated, use of paired- or single-end reads, and other parameters may influence analysis steps and interpretation of the data. Resources to help understand the basics of massively parallel sequencing are provided in S3 Table and the Supplementary Tutorials online (www.rnaseq.wiki). Since most RNA-seq experiments involve comparisons between and across conditions, it is desirable that these factors be consistent across all samples and replicates within an experiment. In addition to classic sources of batch effects (e.g., reagent manufacturing inconsistency), each of these design parameters can introduce systematic biases. Since there is currently a large amount of diversity across published datasets with respect to these and other factors, meta-analyses that combine publicly available data should be pursued with caution. It is likely that RNA-seq will become increasingly standardized with respect to experimental design, data generation, and analysis strategy. Several efforts aimed at establishing best practices are underway (S3 Table). Additional efforts have attempted to characterize the effects of varying specific experimental design factors as well as choice of sequencing platform [4] and the need for technical and biological replicates [53].

## Cloud Computing for RNA-Seq Analysis and Education

To introduce biologists and analysts to RNA-seq analysis techniques, we recommend performing all analyses and tutorials in a cloud-computing environment (e.g., Amazon AWS, Google Cloud, Digital Ocean, etc.). This approach has several advantages for both RNA-seq users and instructors. It ensures a consistent computing environment across all students, and the elasticity of cloud computing allows the same tutorials to be run by small or large groups, even allowing for the possibility of a massive open online course (MOOC). We are able to store machine instances that represent multiple states during the tutorial exercises, including the starting point (a fresh Linux install), an intermediate state with all necessary tools installed, and finally an instance with all analyses complete. These represent useful reference points for comparison to the student’s own work. By performing all tutorials on the cloud, the students learn the basics of cloud computing as they learn about RNA-seq. Using the cloud for instruction also allows the student to easily establish an RNA-seq pipeline in his or her own lab that is based directly on the tutorials, operates in the same environment, and does not require purchasing or administering the substantial hardware that may be needed for RNA-seq data analysis. We provide an extensive introduction to cloud-computing concepts and specific cloud administration skills in the Supplementary Tutorials online (www.rnaseq.wiki). For these tutorials and the following analysis discussions, we selected the “tuxedo” suite and other commonly used tools to illustrate an example RNA-seq analysis workflow. These specific tools were selected because of their widespread use. In our opinion, they are some of the better-engineered options and have acceptable levels of documentation. However, for each analysis application (S1 Table), there are many well-established alternatives (S2 Table) that have merit, and our principal goal was to provide a reference point and help students acquire fundamental skills that will be applicable to other bioinformatics tools and workflows.

## RNA-Seq Data Formats, Quality Control, Trimming, Alignment, and Visualization

In order to understand RNA-seq raw data and alignments, one must develop an understanding of the file formats and underlying data models they represent. These include the FASTA format for storing reference genome data [54], the gene transfer format (GTF) format for storing transcript/gene annotations, the FASTQ format for storing raw read data [55], the sequence alignment map (SAM/BAM) file format for storing read alignments [56], SAM/BAM flags for efficiently classifying certain features of read alignments [56], and compact idiosyncratic gapped alignment report (CIGAR) strings for representing specific linear alignments (S6 Table) [56]. The first steps of RNA-seq workflows often involve some initial quality control (QC) analysis of the raw data in FASTQ files (Fig 5). Without alignments, this typically involves k-mer analysis to identify potential problems such as adapter contamination, inefficient removal of ribosomal sequences, or an abundance of fragments shorter than the target read length. Additional QC metrics obtained at this stage may identify unacceptable base quality profiles, problematic cycles that may have occurred during sequencing, or too many ambiguous bases (indicated as ‘N’ in FASTQ files). Depending on the RNA-seq library construction strategy (S1 Table), some form of read trimming may be advisable prior to alignment of RNA-seq data. Two common trimming strategies include “adapter trimming” and “quality trimming.” Adapter trimming involves removal of the adapter sequence by masking specific sequences used during library construction. Quality trimming generally removes the ends of reads where base quality scores have dropped to a level such that sequence errors and the resulting mismatches prevent reads from aligning. Tools such as skewer [57] and trimmomatic [58] bundle several algorithms together for adjusting raw RNA-seq data and assessing the quality of read data prior to alignment (Supplementary Tutorials at www.rnaseq.wiki). Once read trimming is complete, the next step in most RNA-seq applications is alignment [59] or assembly (Fig 5, S7 Table) [60,61]. RNA-seq assembly is the attempt to merge reads into larger contiguous sequences (contigs) based only on their sequence similarity to each other in the hopes of producing one contig per transcript. This technique does not rely on previously existing reference sequences. RNA-seq alignment involves comparison of each read to a previously assembled reference genome sequence or database of reference transcript sequences. The following discussion and online tutorials mostly assume availability of reference genome and transcript annotations. Once alignments are complete, further QC and interpretation are often desirable prior to downstream analysis applications. RNA-seq alignment data is complex, large, and abstract. These properties create a need for tools and visualization resources that synthesize, summarize, and display raw data in an intuitive interface. Genome browsers such as integrative genomics viewer (IGV) [62], Savant [63], and integrated genome browser (IGB) [64] are capable of displaying RNA-seq alignment files and have been adapted to represent unique features of RNA-seq data such as exon–intron boundaries, splice sites, exon junction read counts, the strand of transcription each read corresponds to, and so on. Some of these browsers have incorporated plug-ins that address specific RNA-seq applications. For example, the “sashimi” plots [65] of IGV allow for the interpretation of complex RNA splicing patterns suggested by coverage patterns and junction spanning reads in an RNA-seq dataset.

## Expression and Differential Expression

One of the most widely used applications of RNA-seq is the estimation of gene or transcript abundance and comparison of these abundances across biological conditions (Fig 5). Gene abundance estimation attempts to measure the transcriptional output for a physical locus in the genome. Transcript abundance estimation deals with the more complex problem of attempting to predict and measure abundance of specific RNA transcript isoforms from each locus. There are two broad strategies for assessing transcript/gene abundance. The first, “count based” method takes the simplifying approach of assigning each read to the single most probable gene based on its alignment location. If the RNA-seq library maintained strand information, this will be used, but in the case of unstranded libraries, only the read position and apparent exon boundaries are used to assign reads to genes. The details of this strategy will depend on whether reads were aligned with a gapped aligner to a reference genome sequence, to a combination of genome and known transcript sequences, or to transcriptome sequences only (S7 Table). In each case, however, the output is a simple integer read count for each gene or transcript. Many methods have been developed to compare these read counts across conditions and to use appropriate normalization strategies and statistical tests to determine which genes are differentially expressed (S1 Table and S2 Table) [66–68].

While the raw read count methods are well developed and have been validated as a robust alternative to expression microarrays and gold standard assays such as qPCR [69], they have the caveat that it is difficult to use this output to compare gene expression estimates within a single sample or to assess which genes are expressed above background noise levels. Raw read counts are most valid when they are compared across multiple samples processed identically. The reason for this is that additional factors other than abundance influence the read count expected for each gene. For example, a gene may have a higher read count simply by being larger. Similarly, sequencing bias related to guanine and cytosine (GC) content and other factors may skew read counts for each gene. Methods such as Cufflinks attempt to obtain an abundance measure that is useful in an absolute sense as well as the relative sense described above [8]. The “FPKM” (fragments per kilobase of transcript per million mapped reads) measure of Cufflinks and other tools (S2 Table) attempts to obtain an abundance estimate and associated confidence interval for each gene and transcript/isoform (S7 Table). The abundance of each transcript is estimated with a maximum likelihood probabilistic model that makes use of information such as fragment length distribution, gene size, GC content, number of multimapping reads, and the number and structure of predicted isoforms. This is a much more complicated problem than assigning a simple read count and is an active area of research (http://biorxiv.org/content/early/2014/07/14/007088).

In order to estimate transcript abundance, Cufflinks first builds transcript isoforms by identifying overlapping “bundles” of fragment alignments. These are assembled, fragments are connected in an overlap graph, and transcript isoforms are inferred from the minimum paths required to cover the graph. Following use of Cufflinks to estimate transcript structures and abundance in each sample, Cuffmerge is used to merge several Cufflinks assemblies together (Fig 5). This is necessary because, even with replicates, Cufflinks will not necessarily assemble the same numbers and structures of transcripts in each sample. Cuffmerge also removes a number of transfrags (short transcript predictions) that are probably artifacts. Using Cuffmerge, one can make an assembly GTF file suitable for use with Cuffdiff [14] to generate and compare abundances for a unified transcriptome model across several samples. In Cuffdiff, the variability in fragment count for each gene across replicates is modeled (Fig 5). The fragment count for each isoform is estimated in each replicate, along with a measure of uncertainty in this estimate arising from ambiguously mapped reads. Transcripts with more shared exons and few uniquely assigned fragments will have greater uncertainty. The algorithm combines estimates of uncertainty and cross replicate variability under a beta negative binomial model of fragment count variability to estimate count variances for each transcript in each library. These variance estimates are used during statistical testing to report significant differentially expressed genes and transcripts. In the Supplementary Tutorials (www.rnaseq.wiki), we explore the use of both count based and “FPKM style” expression estimation and associated differential expression tools.

Interpretation, summarization, and visualization of expression and differential expression results can be just as involved as generating these results (Fig 5). CummeRbund [15] accepts Cuffdiff output and automatically generates many useful data visualizations. There are many additional resources for downstream interpretation of the biological significance of expression and differential expression results (S2 Table and S8 Table). In the tutorials accompanying this work, we provide guidance on how to format Cufflinks data and start manipulating it with R (http://www.R-project.org) and Bioconductor [70]. While we still rarely have sufficient sample size and clinical details for classification exercises, these data are becoming more available. We recommend Weka [71,72] as a good learning tool and the RandomForests R package for robust classifier building. Similarly for pathway and gene set analysis, we recommend SeqGSEA (sequence based gene-set enrichment analysis) [73], GAGE (generally applicable gene-set enrichment for pathway analysis) [74], PathView [75], GoSeq (gene ontology analysis for RNA-seq) [76], GSAASeqSP (gene-set association analysis for RNA-seq with sample permutation) [77], and Cytoscape [78,79].

## Isoform Discovery and Alternative Expression

**Fig 6:**
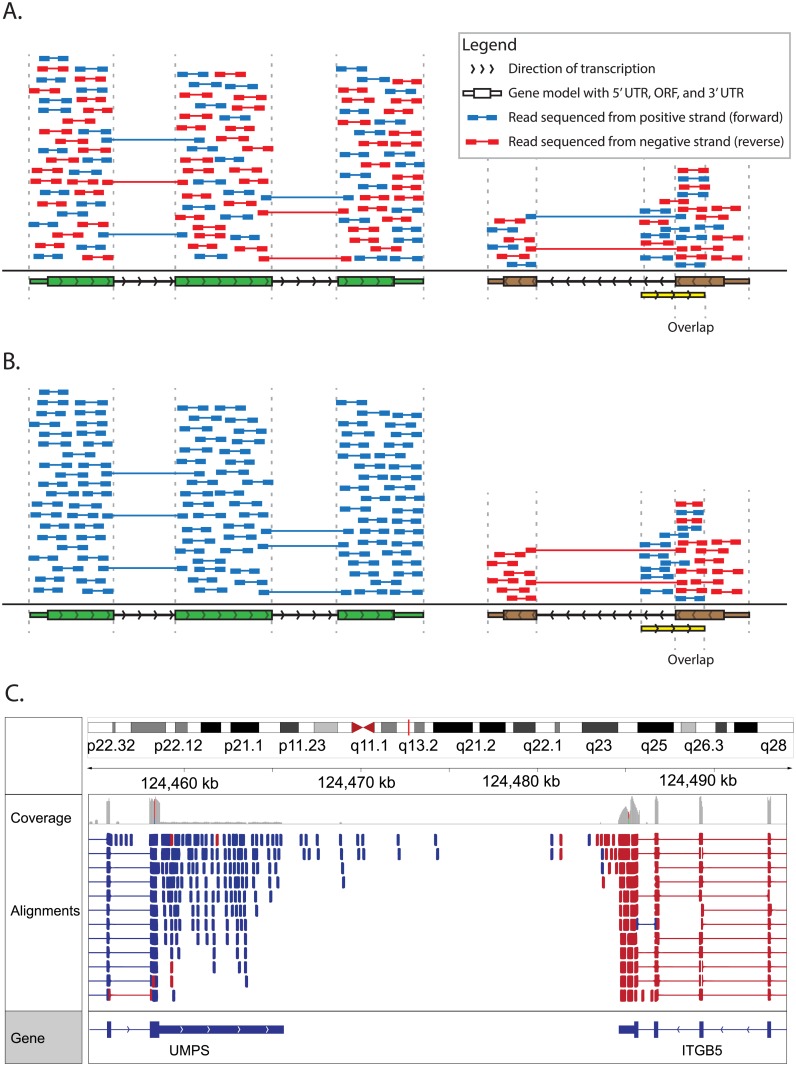
Comparison of stranded and unstranded RNA-seq library methods and their influence on interpretation and analysis. (A) Many RNA-seq library construction protocols do not maintain the strand identity of RNA transcripts in the sequence data (S1 Table). In these “unstranded” strategies, double-stranded cDNA is sequenced, and knowledge of the transcription strand of the RNA molecule is lost. This results in an even mix of reads from both strands. In panel A, a gene transcribed on the positive strand is shown in green, a second gene transcribed on the negative strand is shown in brown, and a third gene transcribed on the positive strand (partially overlapping the second gene) is shown in yellow. The first two genes are protein coding with the open reading frame (ORF) portion depicted as thick rectangles and the UTRs depicted as thin rectangles. The third gene is a noncoding RNA gene. Aligned paired-end read sequences (read 1 and read 2) are depicted as short colored bars connected by thin lines. The thin connecting line in each read pair depicts the portion of the cDNA fragment that remains unsequenced when the cDNA fragment is larger than two times the read length. Each read is colored according to the strand sequenced, blue for the positive (forward/sense) strand and red for the negative (reverse/antisense) strand. Using known annotations, the mapped position of each read, and knowledge of exon splicing patterns, the likely transcription strand of some reads can be inferred. However, for many aligned reads the transcription strand cannot be inferred and sense-antisense expression analysis is not possible. Note that for each gene, an approximately equal proportion of reads corresponding to each strand are observed. Also note that read pairing information can sometimes be used to infer which gene a read was likely derived from. These reads are referred to as “encompassing” read pairs, in which one read of a pair aligns within one exon and the second read of a pair aligns within another exon. However, reads that align within a region corresponding to overlapping genes cannot be unambiguously assigned to either gene (e.g., the portion of the brown and yellow genes that overlap). Note that in this figure we are not depicting any reads in which a single read of a read pair spans across an intron. These exon–exon “spanning” reads can usually be matched unambiguously to a transcript, even in an unstranded library, because the exon–exon junction alignments line up with known splice sites and exon boundaries. (B) More recent “stranded” RNA-seq library strategies allow the strand information to be retained. In the resulting alignments, depicted in panel B, the strand of the alignment corresponds in a predictable way to the transcription strand of the sequenced RNA molecule. Now we see that reads aligning within a gene are indicated as being derived from the expected transcription strand for that gene. Furthermore, in regions where two genes overlap on opposite strands, we can now unambiguously assign reads to each gene. (C) When strand information is maintained by the RNA-seq protocol, it can be visualized in genome browsers such as IGV [62]. For example, to make IGV color read alignments according to strand, use the “Color alignments” by “First-of-pair strand” setting (refer to S5 Table for more strand-related software settings).

While many RNA-seq experiments focus on abundance estimation and differential expression analysis of known genes, the relatively unbiased “shotgun” sampling of RNA-seq also allows for discovery of novel transcript isoforms, detection of differential splicing patterns, and detection of chimeric fusion genes. However, these applications are limited by the considerable challenges associated with inferring full-length transcripts from relatively short RNA-seq fragments. The average human protein coding transcript has ~8–10 exons and is ~2,000 bp in length. However, an RNA-seq library is made up of fragments of cDNA of ~200–400 bp in length that are only partially sequenced from each end. Furthermore, the strand from which the original mRNA sequence was transcribed is unknown in many library preparation strategies, though sometimes we can infer the strand by examining splice site spanning reads (Fig 6). We can also infer local structural information about the transcript a cDNA fragment may have been derived from. Cufflinks and its competitors (S2 Table) provide sophisticated modeling for such inferences, but the underlying problem being addressed is very complicated. The larger the transcripts and the more transcripts expressed from a single locus, the harder it is to determine full-length transcript sequences and their abundance. Each transcript isoform has very few (if any) exons and exon–exon junctions that are unique to that isoform. Some approaches [3,80], sidestep this complexity by focusing on individual sequence features without attempting to infer the structure of full-length transcripts. This simplifies the problem to identifying alternatively expressed exons or junctions that can then be studied in the lab with a technique more suitable to resolving full-length cDNA structures. The gold standard remains full-length sequencing of a large pool of cloned cDNAs generated by RT-PCR [81,82]. This is labor intensive, and RNA-seq remains a viable interim strategy for transcriptome-wide alternative expression analysis. We provide two example workflows in the Supplementary Tutorials and additional resources to help the reader explore additional alternatives (S1 Table and S2 Table).

## Challenges Specific to RNA-Seq

There are several challenges that are specific to RNA-seq analysis [83] compared to DNA-level analysis. Foremost among these are issues relating to sample purity, quality, and quantity. RNA is unstable and prone to degradation, requiring many specialized QC, sample handling, and analysis strategies (S3 Table and S7 Table). Sample QC is commonly determined by capillary electrophoresis of total RNA on a platform that provides a semiquantitative estimation of RNA integrity (see Fig 3 and S1 Data). The construction of RNA-seq libraries for sequencing has changed rapidly since its adoption, and associated variations in library preparation can influence RNA-seq study design, analysis, and interpretation. This includes variations in RNA isolation and storage methods, strategies for RNA enrichment, fragmentation and size selection methods, the use of amplification, the maintenance of transcript strand identity, library normalization, sample indexing, and more (S1 Table). Compared to DNA sequence analysis, the read alignment stage of RNA-seq is considerably more challenging [84]. In eukaryotes, the need to resolve exon/intron structure from relatively short reads complicates alignment and downstream analysis steps. Exons can be separated by large introns such that a single sequence read alignment might span hundreds of kilobases across two or more gaps corresponding to intron splice sites. Furthermore, compared to genome sequencing, the expected relative abundance of RNAs vary widely, with published estimates suggesting that at least 10^5^–10^7^ orders of magnitude are expected between genes with the lowest and highest expression [85,86]. Since RNA-seq works by random sampling, a small fraction of highly expressed genes can consume the majority of reads. One consequence of this wide range is that in order to capture a snapshot of the transcriptome that includes lowly expressed genes, an RNA-seq library must be much deeper than one might expect based on the proportion of bases in a genome that are annotated as expressed. Ribosomal and mitochondrial genes are particularly highly expressed in many tissues, and important steps in both RNA-seq library preparation and analysis strategies are concerned with removing them or the biases related to them [87]. Another distinct feature of RNA molecules that affects analysis is that they occur in a wide range of sizes. Very small RNAs (<100bp) such as micro RNAs (miRNA) must generally be captured and sequenced by an independent strategy, as size selection strategies would normally exclude these (Fig 3) [88,89].

## Conclusions and Future Work

Certain questions consistently arise among researchers performing RNA-seq analysis. To avoid repetition of effort, we advocate for these questions to be asked and answered within “BioStars” (www.biostars.org), an online question-and-answer forum for bioinformatics [90] in which a community can improve and update answers as RNA-seq analysis practices evolve. In S7 Table, we provide answers to many common questions that cover topics such as whether to remove duplicate reads, how to select replicates, and the target depth of sequencing to perform.

The analysis goals of RNA-seq experiments are diverse. Each of these analysis goals has distinct requirements and challenges. However, a common workflow generally involves obtaining raw data, preprocessing this data and performing basic quality assessment, either aligning or assembling reads, processing the resulting alignments with a tool specific to the analysis goal, postprocessing custom output files from this tool, and summarizing and visualizing the final results (Fig 5). In the supplementary materials, we reference specific resources (S2 Table and S8 Table) relevant to each of these steps. However, we focus on the basics of RNA-seq data analysis that are common to all applications as described above, followed by detailed consideration of reference-guided transcriptome assembly, transcript quantification, differential expression, and alternative expression. We provide documented RNA-seq analysis pipelines to allow hands-on demonstration of some of these analysis goals (Supplementary Tutorials at www.rnaseq.wiki) using example data sets (S2 Data). We will continue to expand these resources to cover additional applications as we use this content at various educational workshops offered through the Canadian Bioinformatics Workshops (CBW), Cold Spring Harbor Laboratories (CSHL), and future collaborating partners. We recognize that the current example workflow relies heavily on the existence of a reference genome sequence. We hope in the future to add example workflows that use reference-free or alignment-free methods of RNA quantification (see S2 Table for example tools). We also hope this work will help other groups create new RNA-seq pipelines and improve existing RNA-seq education initiatives (S9 Table). All materials described here are released in a freely available, version-tracked, open-access format under a Creative Commons license at www.rnaseq.wiki.

## Supporting Information

See supplementary materials section and the online wiki that accompanies this article: www.rnaseq.wiki. Additional references are provided in S1 Text.

## Supporting Information

S1 Data “Database” of Agilent examples as a resource to assist interpretation of RNA integrity numbers (RINs). (PDF)

S2 Data Data used in the online tutorial for RNA-seq analysis. (PDF)

S1 Table RNA-seq analysis techniques. There are several downstream analysis goals for which RNA-seq is well suited. The main categories of these are described briefly below with reference to supporting materials. Refer to S2 Table for specific tools relevant to many of these areas. For each application, a basic data recommendation is provided. It is important to remember that these are simply examples. In addition to the varying demands of each analysis technique, data requirements will depend heavily on the size and complexity of the genome, the complexity of the transcriptome, the method of RNA isolation and library preparation, the need to robustly detect transcripts with low copy numbers, and many other factors. For the purposes of this table, low RNA-seq depth is 5–25 million reads, moderate depth is 25–100 million reads, and high depth is 100–500 million reads. Similarly, short reads are 50–200 bp, and long reads are 200–500 bp.
(PDF)

S2 Table Tools for RNA-seq analysis. All tools used in the online tutorial (www.rnaseq.wiki) are referenced below (tool name in bold) along with alternative tools in each category. Whenever possible, a citation is provided. Links are also provided to help the user evaluate the code and the level of maintenance. Whenever possible, the link goes directly to a source controlled repository such as a git repo. Additional lists of tools can be found here: Alamancos et al. (arXiv), the rna-seqblog, and RNA-seq—Protocols and Algorithms. This table is meant to be comprehensive but not exhaustive. Some RNA-seq analysis topics that are not explicitly covered here include co-regulation (co-expression), disease classification, time series, expression compendium databases, outlier expression, data normalization, and miRNA analysis.
(PDF)

S3 Table Concepts in sample preparation and library construction that can influence study design, analysis, and interpretation. The following table summarizes several key concepts relating to sample preparation and library construction that may influence analysis and interpretation of RNA-seq data. Several initiatives are underway to develop standards and best practices that cover many of these concepts. These include the Sequencing Quality Control (SEQC) consortium, the Encyclopedia of DNA Elements (ENCODE) consortium, the Roadmap Epigenomics Mapping Consortium (REMC), and the Beta Cell Biology Consortium (BCBC).
(PDF)

S4 Table Description of RNA-seq library enrichment strategies. A description of three RNA enrichment strategies is provided, along with their anticipated effects on RNA-seq library construction and data interpretation. For a visual depiction of the concepts discussed here, refer to Fig 4.
(PDF)

S5 Table Strand-related settings for RNA-seq tools that must be adjusted to account for library construction strategy. This table provides further explanation of IGV’s read orientation codes for RNA-seq data viewed in the browser. Also provided are recommended software settings for three additional tools involved in common RNA-seq analysis workflows: TopHat, HTSeq, and Picard. Each of these explanations/settings is provided for several commonly used RNA-seq library construction kits that produce either stranded or unstranded data.
(PDF)

S6 Table Standard file formats and tool-specific files used in RNA-seq analysis. The following table describes several file formats used in most RNA-seq analysis workflows as well as several files specific to the expression analysis tools used by the online tutorials that accompany this article (at www.rnaseq.wiki).
(PDF)

S7 Table Common RNA-seq analysis questions and their answers. The following table summarizes a list of commonly asked questions relating to RNA-seq analysis, with links to BioStar posts in which these questions have been addressed by the community.
(PDF)

S8 Table General resources for RNA-seq analysis. The following table provides a list of general resources to help understand the background of RNA biology, next-generation sequencing, RNA-seq laboratory methods, and RNA-seq analysis. Additional educational resources can be found in the resources section of the online tutorial at www.rnaseq.wiki.
(PDF)

S9 Table RNA-seq workshops and online tutorials. The following table lists RNA-seq workshops and other tutorials complementary to this article. These examples are limited to online materials or short workshops. Not listed here are formal training programs or degrees in bioinformatics. For ongoing discussion of this topic, refer to these BioStar posts: https://www.biostars.org/p/79845/ and https://www.biostars.org/p/11034/.
(PDF)

S1 Text Supplementary references. (PDF)
