# Supplementary material for: Informatics for RNA Sequencing: A Web Resource for Analysis on the Cloud
Source: PLoS Comput Biol. 2015 Aug 6;11(8):e1004393. doi: 10.1371/journal.pcbi.1004393 (PMC4527835; doi:10.1371/journal.pcbi.1004393)
Supplement: S1 Data — (PDF) [file pcbi.1004393.s001.pdf]

S1 Data 1. 'Database' of Agilent examples as a resource to assist interpretation of RIN numbers.
Agilent Trace Examples

For further explanation of the RIN scoring system, refer to:
Agilent Trace Intro
Agilent Traces for Total RNA
Agilent Traces for mRNA
