# Supplementary material for: Informatics for RNA Sequencing: A Web Resource for Analysis on the Cloud
Source: PLoS Comput Biol. 2015 Aug 6;11(8):e1004393. doi: 10.1371/journal.pcbi.1004393 (PMC4527835; doi:10.1371/journal.pcbi.1004393)
Supplement: S2 Data — (PDF) [file pcbi.1004393.s002.pdf]

S2 Data. **Data used in the online tutorial for RNA-seq analysis.**
[Description of raw data used in the tutorials]
[FTP site for raw data]

The test data consists of two commercially available RNA samples: Universal Human Reference (UHR) and Human Brain Reference (HBR). The UHR is total RNA isolated from a diverse set of 10 cancer cell lines. The HBR is total RNA isolated from the brains of 23 Caucasians, male and female, of varying age but mostly 60-80 years old.

In addition, a spike-in control was used. Specifically we added an aliquot of the ERCC ExFold RNA Spike-In Control Mixes to each sample. The spike-in consists of 92 transcripts that are present in known concentrations across a wide abundance range (from very few copies to many copies). This range allows us to test the degree to which the RNA-seq assay (including all laboratory and analysis steps) accurately reflects the relative abundance of transcript species within a sample. There are two 'mixes' of these transcripts to allow an assessment of differential expression output between samples if you put one mix in each of your two comparisons. In our case, Mix1 was added to the UHR sample, and Mix2 was added to the HBR sample. We also have 3 complete experimental replicates for each sample. This allows us to assess the technical variability of our overall process of producing RNA-seq data in the lab.

For all libraries we prepared low-throughput (Set A) TruSeq Stranded Total RNA Sample Prep Kit libraries with Ribo-Zero Gold to remove both cytoplasmic and mitochondrial rRNA. Triplicate, indexed libraries were made starting with 100ng Agilent/Stratagene Universal Human Reference total RNA and 100ng Ambion Human Brain Reference total RNA. The Universal Human Reference replicates received 2 ul of 1:1000 ERCC Mix 1. The Human Brain Reference replicates received 1:1000 ERCC Mix 2. The libraries were quantified with KAPA Library Quantification qPCR and adjusted to the appropriate concentration for sequencing. The triplicate, indexed libraries were then pooled prior to sequencing. Each pool of three replicate libraries were sequenced across 2 lanes of a HiSeq 2000 using paired-end sequence chemistry with 100bp read lengths.
