# Supplementary material for: Informatics for RNA Sequencing: A Web Resource for Analysis on the Cloud
Source: PLoS Comput Biol. 2015 Aug 6;11(8):e1004393. doi: 10.1371/journal.pcbi.1004393 (PMC4527835; doi:10.1371/journal.pcbi.1004393)
Supplement: S1 Table — There are several downstream analysis goals for which RNA-seq is well suited. The main categories of these are described briefly below with reference to supporting materials. Refer to S2 Table for specific tools relevant to many of these areas. For each application, a basic data recommendation is provided. It is important to remember that these are simply examples. In addition to the varying demands of each analysis technique, data requirements will depend heavily on the size and complexity of the genome, the complexity of the transcriptome, the method of RNA isolation and library preparation, the need to robustly detect transcripts with low copy numbers, and many other factors. For the purposes of this table, low RNA-seq depth is 5–25 million reads, moderate depth is 25–100 million reads, and high depth is 100–500 million reads. Similarly, short reads are 50–200 bp, and long reads are 200–500 bp. (PDF) [file pcbi.1004393.s003.pdf]

S1 Table. RNA-seq analysis techniques

There are several downstream analysis goals for which RNA-seq is well suited. Main categories of these are described briefly below with reference to supporting materials. Refer to **S2 Table** for specific tools relevant to many of these areas. For each application, a basic data recommendation is provided. It is important to remember that these are simply examples. In addition to the varying demands of each analysis technique, data requirements will depend heavily on the size and complexity of the genome, the complexity of the transcriptome, the method of RNA isolation and library preparation, the need to robustly detect transcripts with low copy numbers, and many other factors. For the purposes of this table, low RNA-seq depth is 5-25M reads, moderate depth is 25-100M reads, and high depth is 100-500M. Similarly, short reads are 50-200bp and long reads are 200-500bp.

**RNA-seq analysis technique**	**Description**
Gene annotation and transcript discovery [7-13]	RNA-seq produces short reads (often paired) from short (~100-500 bp) fragments of cDNA by shotgun sequencing. When performed to high depth for a single sample it is possible to make strong inferences regarding the regions of transcriptional activity within a genome and the exon-intron structure of transcripts expressed from each region. When a reference genome sequence is available, RNA-seq reads can be aligned to this sequence using a splice aware aligner and the intron-exon boundaries and exon-exon connections of expressed transcripts can be determined. More generally, the gene loci where expression occurs can be enumerated. The presence of multiple transcript isoforms expressed from a single locus can also be determined in many cases, though the full length structure may be difficult to completely resolve. In addition to aligning reads to a reference genome sequence, transcripts can also be inferred by *de novo* transcript assembly. If a reference genome sequence is available, the resulting assembled contigs can be aligned and used to determine exon-intron structure. If no reference genome sequence is available, the resulting contigs can still be used for gene annotation by analysis with ORF finding tools, sequence conservation comparisons to related species, etc.<br>*Data recommendation*: This application will benefit from longer reads, especially in species with large introns, small exons, and complex splicing patterns. Sequence depth will influence the comprehensiveness of transcripts that can be annotated, with lowly expressed transcripts requiring perhaps 100M reads or more to effectively cover.
Gene and transcript expression estimation [91-94]	In the 'gene annotation and transcript discovery' discussion above we described the use of RNA-seq to determine which gene loci are transcribed by RNA polymerase and how the exon-intron structure of those transcripts is determined by the splicing machinery. Gene and transcript expression estimation by RNA-seq involves the abundance estimation of all transcripts or individual transcript isoforms expressed

	from each locus. This step often relies on existing transcript annotations for a species (e.g., a transcriptome GTF file from Ensembl) or it requires that you first predict the transcripts present in your data and then derive abundance estimates for those transcripts. Cufflinks and HTSeq are two examples of tools used to estimate transcript and gene abundances. To estimate abundance by RNA-seq, reads are aligned either to a reference genome, reference transcript sequences, assembled transcript contigs derived from the same data, or some combination of these. The read count observed at each locus or for each known transcript sequence is then used to estimate the relative abundance of each transcript. If a spike-in reagent with known concentrations was used during library construction, it may be possible to estimate absolute copy number values in the sample. Abundance estimation tools may attempt to normalize expression values to account for biases related to the different sizes of transcripts, varying GC content, varying library sequence depth, and so on. Further normalization across a series of samples may involve examination of a set of 'housekeeping genes' and/or use of other data normalization techniques [95].<br>*Data recommendation*: This application places one of the lowest demands on library depth compared to other RNA-seq analysis techniques. For gene-level expression estimation, as few as 5-10M reads may be sufficient for mRNA-seq libraries and where possible additional replicates may be preferable to deeper individual libraries. This application will work well with long or short reads that may or may not be paired end.
Differential gene or transcript expression analysis [15-17]	Differential gene or transcript expression involves comparison of abundance estimates between two or more conditions. For example, the abundance of a gene observed in different tissues, developmental stages, chemical exposures, disease versus healthy states, etc. There are many biases that influence abundance estimates for each gene or transcript. One advantage of differential expression analysis is that many of these biases will be consist across the samples and 'cancel out' leaving potentially biologically relevant differences in gene expression. Unfortunately, many factors may introduce systematic bias that is not equal across the RNA-seq data sets being studied. There are many approaches for identifying batch effects and for performing data normalization that may mitigate the effect of these systematic biases.<br>*Data recommendation*: This application has close to the same data requirements as expression estimation except that some additional sequence depth may be required to accurately estimate subtle differences in expression between samples.
Alternative expression (alternative transcript initiation, polyadenylation, and splicing)	Alternative expression is closely related to differential expression but attempts to identify differences in the relative ratios of alternative isoforms expressed from a locus. It is possible for the overall expression output from a locus or to remain unchanged between two conditions but have a significant shift in the relative expression levels of alternative isoforms. Alternative expression can be caused by changes in the use of alternative transcript initiation sites, exon-intron splice sites, and polyadenylation

analysis [3]	sites at a locus. Many human protein-coding loci have extensive potential for alternative expression and the majority of human loci have at least one known alternative isoform. Subtle changes in the structure of transcripts may have pronounced functional consequences but have relatively subtle effects on transcript or gene abundance estimates. Alternative expression analysis by RNA-seq has the potential for a more nuanced representation of the transcriptional state of a sample compared to simple gene expression analysis, though it comes at the cost of more complicated algorithms.<br>*Data recommendation*: This application will place some of the highest demands on library depth and read length. To robustly assay the alternative expression patterns of human tissues we recommend at least 300-500M reads. This application will also benefit from longer reads.
Allele specific expression analysis (ASE) [18, 19]	In diploid (or polyploid) species RNA expression can occur independently from each inherited chromosome. Maternal and paternal derived alleles of each gene locus may contain sequence differences such as common polymorphisms (e.g., SNPs) and mutations. They also differ in their methylation (e.g., imprinting) or other epigenetic states. While many gene loci exhibit balanced expression from each allele, some loci exhibit unbalanced or allele specific expression patterns [18]. This allele specific bias could be caused for example by a polymorphism near a promoter that increases transcription factor recruitment and increased polymerase activity for one allele compared to another. The same kinds of allele specific effects can influence choice of alternative transcript initiation sites, alternative splice sites, and alternative polyadenylation sites. Mutations can result in completely novel expressed isoforms being generated from the mutated allele. Allele specific expression analysis uses the presence of known heterozygous polymorphisms within the expressed portion of genes to observe the balance/imbalance of expression from both alleles. In order to perform allele specific expression analysis it is desirable to accurately identify these heterozygous sites in the individual being studied. One therefore typically needs both DNA sequence (DNA-seq) (e.g., WGS or Exome) data as well as RNA-seq data for each sample to be analyzed for allele specific expression.<br>*Data recommendation*: This application has moderate demands for library depth compared to other RNA-seq analyses. Getting accurate variant allele frequencies (VAFs) will require low library sequence depth for highly expressed genes but high library sequence depth for lowly expressed genes. Measuring allele specific expression for single nucleotide variants will work well with 100 bp reads (or perhaps shorter). Measuring allele specific expression for insertions and deletions (especially >10-20 bp) will benefit from longer read length libraries.
RNA editing analysis [20-22]	RNA editing describes nucleotide sequence modifications to RNA molecules that happen after transcription by an RNA polymerase. Such modifications result in apparent changes in the RNA sequence from that which would be predicted from the genome sequence. It is possible to

	detect such sequence changes in RNA-seq data, but in order to be convinced that the change is due to RNA editing, DNA-seq data is required for the same sample. In simple terms, by comparing the transcribed sequence by RNA-seq to the genome sequence by DNA-seq (WGS or Exome) one can infer that RNA editing has taken place at the RNA level. However, due to sequence errors, mapping artifacts and other sources of systematic biases, care must be taken to distinguish false positives from true RNA editing events and the prevalence of RNA editing as determined by RNA-seq analysis remains a controversial area of research.<br>*Data recommendation*: This application has moderate demands for library depth, similar to those for allele specific expression. However, since RNA-edits consist primarily of single nucleotide changes, longer read lengths are a lower priority for this application compared to allele specific expression analysis.
Variant detection (variant discovery) [31-33]	While variant detection typically involves analysis of DNA-seq data such as WGS or exome data [96], it is also possible to perform detection of single nucleotide variants and small insertions or deletions using RNA-seq data [31-33]. RNA-seq variant detection involves alignment of RNA-seq reads to a reference genome sequence or database of reference transcript sequences followed by scanning the resulting alignments for sites that exhibit sequence base differences relative to the reference sequences. The proportion of reads harboring the variant sequence is used to calculate a variant allele frequency (VAF) from 0 to 100%. The number of variant supporting reads, VAF, base qualities at the variant position, read alignment qualities, overall level of coverage, and other factors collectively influence the confidence of each variant prediction (i.e. the probability that it is a real variant and not a false positive). There are several factors that complicate this variant detection when performed with RNA-seq instead of DNA-seq data. In eukaryotic species, the presence of introns complicates alignment of reads to a reference genome and may lead to alignment errors. In some cases, these errors may result in false positive variant calls where repeated alignment errors result in systematic mismatches. These false positives are enriched near the edges of exons when performing variant discovery with RNA-seq data because correctly resolving exon-intron-exon alignments is difficult, especially with large introns and where only a short portion of a read spans from one exon to the next. Reads that mostly align to one exon but spill over the edge of it can result in misaligned bases. False negatives may also occur in regions of the genome that are difficult to map to, and these alignment holes may be more prevalent where exon-intron structures complicate alignment. Some of these alignment issues may be overcome by aligning reads directly to predicted transcript sequences and performing variant detection by observing sequence differences between the known transcript sequence and aligned reads. Library end bias and corresponding lack of coverage near the 5’ end of transcripts may also result in false negatives due to poor coverage. Furthermore, detection of

	polymorphisms will be limited to genes that are expressed in the tissue being profiled and the ability to call variants within expressed genes will vary across the range of expression levels. In highly expressed genes, coverage may be extremely high. This can lead to detection of false positives at low variant allele frequency if the variant detector does not use appropriate statistics. In genes that are not expressed, variant detection will not be possible. Some mutations within exons may lead to nonsense mediated decay (NMD) that results in decreased stability of the mutant harboring transcripts. This will reduce the ability to detect such loss of function events when using RNA-seq data alone.<br>*Data recommendation*: This application has moderate to high demands on library depth and read length depending on the specific type of variant detection as outlined in the following four entries of this table.
Common polymorphism detection [31-33]	It is possible by RNA-seq analysis to detect common polymorphisms (e.g., SNPs) that occur within expressed exons [31, 32]. The sites of many of these are known in many species and this knowledge can be used to guide their detection. Since the expected frequency of heterozygous and homozygous polymorphisms is high (~50% and ~100% respectively) they can be readily detected even in genes with low expression levels and therefore low read coverage. As discussed above, allele specific expression may reduce or increase the expected frequency of heterozygous SNPs. Since the majority of common polymorphisms occur within introns or outside of gene loci, a relatively narrow subset of polymorphisms will be assayed by RNA-seq data alone.<br>*Data recommendation*: This application has moderate demands on library depth. Since the variants are expected to occur at 50 or 100% VAF, detecting them should be possible with 20-30x coverage at each site. Target library depth will be driven by the amount of data needed to achieve this coverage for lowly expressed genes. As described for allele specific expression above, variants with substantial nucleotide differences from the reference genome sequence (e.g., insertions and deletions) may benefit from longer read lengths to facilitate accurate alignment of reads containing the variant sequence.
Germline mutation detection [31-33]	RNA-seq analysis for germline mutation detection is largely equivalent to the detection of polymorphisms as described above except the variants being discovered are very rare in the population (they may even be private to a single individual). Without prior knowledge of the expected site of mutation the analysis must scan the entire transcriptome. Such analysis may be greatly aided by having RNA-seq data from related family members (e.g., a trio of mother, father, child). As with polymorphism detection, mutation detection in RNA-seq data will be complicated by the varying expression levels of each gene and allele specific expression.<br>*Data recommendation*: This application has essentially the same data needs as common polymorphism detection described above.
Somatic mutation	Somatic mutation detection has many similarities to other types of variant detection described above. It still involves detection of variants but adds

detection [31-33]	an extra consideration to identify the subset of variants that were likely acquired in the DNA of the tumor (i.e. those that are not germline inherited variants). Somatic mutation detection is possible but difficult with RNA-seq data compared to DNA-seq data such as WGS or exome data. Using DNA-seq data it is common to compare tumor sequence data directly to matched normal data to assess the somatic status of variants. The normal DNA sample is usually blood in the case of solid tumors, and usually a skin biopsy in the case of hematologic tumors. Since we expect approximately even coverage across the genome (or exome) for both the tumor and normal sample, we can compare DNA-seq reads at each position harboring a variant in the tumor data and assess its presence in the normal data. Convincing somatic variant sites will have good sequence coverage in both the normal and tumor sample but will only have significant support for the variant base in the tumor data. This kind of sample pairing for tumor/normal comparison is not usually appropriate for RNA-seq data. Using a blood normal RNA sample as a comparator for a solid tumor would not work well because the gene expression pattern for the solid tumor would not be expected to match that of the blood sample. In other words we often may not have coverage of variant sites in both normal and tumor. Furthermore, there may be differences in allele specific expression between the tumor and normal comparator. For some tumor types, it may be possible to obtain a tissue-matched normal sample to use as a comparator for determining somatic status. For example, a breast tumor sample could be compared to adjacent normal breast tissue obtained from the same individual. However, even in such cases, the matched normal may not have the same composition of cell types that the tumor has and there may be significant differences in the transcriptome landscape between tumor and normal samples that confounds somatic variant determination. One strategy that could be used to circumvent this challenge is to compare the tumor RNA-seq data to normal DNA-seq data such as exome data. Given the decreasing cost of WGS and exome data it is probably more appropriate to simply produce RNA-seq data for the tumor and DNA-seq data for both the tumor and a matched normal.<br>*Data recommendation*: This application is similar to other variant detection types but has substantially increased demands on library sequence depth compared to other categories because it involves the detection of somatic variants in tumor samples that may be contaminated with normal DNA (thereby reducing the observable VAF and number of variant supporting reads) or confounded by tumor heterogeneity (where some mutations exist only in subclonal populations). If a normal RNA sample is used as a comparator to determine the somatic status (often not possible), good coverage of that sample will also be required. As with other variant detection types, characterization of complex variants may benefit from longer reads.
Mutation expression	Perhaps the most common application of RNA-seq data in the sphere of mutation detection is to first detect all mutations (germline or somatic)

assessment [97]	using DNA-seq data and then only use the RNA-seq data to assess the expression status of each mutation [97]. This is equivalent to the allele specific expression analysis described above except that instead of relying on known sites of polymorphism common in the population it relies on a prior mutation detection step using DNA-seq data. The ability to assess the expression status of mutations varies by the complexity of the mutation. Single nucleotide variants (SNVs) will be relatively straightforward but larger insertions and deletions may be more challenging due to challenges in alignment. In general, one should be careful to remember that the failure to confirm expression of a mutation observed at the genome level in the transcriptome could be influenced by differences in alignment between the DNA-seq and RNA-seq at the site of the mutation as well as other RNA-seq specific biases such as reduced RNA-seq coverage at the 5' ends of transcripts.<br>*Data recommendation*: This application has perhaps the lowest demands on library depth compared to other variant detection applications since the variants are already detected at the DNA-level and RNA-seq data is only used to assess their expression level. However, these variants may occur anywhere in a transcript and might occur in transcripts will low but functionally significant expression levels. In other words, comprehensive and deep coverage of the transcriptome is still desirable for this application. As with other variant detection types, characterization of complex variants may benefit from longer reads.
Gene fusion detection [26-30]	Gene fusion detection by RNA-seq is mostly performed in the context of tumor sequencing projects [26, 98, 99]. A gene fusion is a chimeric transcript that combines portions of two transcripts normally expressed from two distinct gene loci, 'gene A' and 'gene B' (e.g., *BCR-ABL, EML4-ALK*, etc.). Gene fusions may arise as a consequence of structural variations in the genome such as deletions, insertions, inversions, and translocations. Identification of fusion events in RNA-seq data relies on two main forms of alignment information. (1) Paired-end read information where one read of a pair maps to 'gene A' and the other read of that pair maps to 'gene B'. Such reads are sometimes referred to as encompassing reads. (2) Individual reads that align across the junction of 'gene A' and 'gene B'. For example, a read where the first half maps to the edge of an exon in 'gene A' and the second half of this read maps to the edge of an exon in 'gene B'. Such reads are sometimes referred to as spanning reads. Drops or spikes in read coverage levels across the length of either 'gene A' or 'gene B' that correspond to the apparent breakpoint may also help to support the existence of a gene fusion. In some cases, 'soft clipped' reads that align partially and become stretches of mismatches may suggest the presence of a fusion breakpoint. Fusion detection tools currently under development attempt to combine evidence from both the RNA and DNA level to give more accurate predictions.<br>Gene fusion detection tools generally have complex processes for producing alignments suitable for fusion detection, filtering steps to remove false positives that occur widely in genes with paralogs, an

	assembly step that attempts to determine the fusion sequence, annotation steps that attempt to determine if an in frame fusion product is likely to result from an RNA fusion transcript, and additional annotation steps.<br>*Data recommendation*: This application has moderate demands on library depth assuming that an oncogenic fusion gene is likely to be expressed at reasonably high levels. Reads should be paired-end as most fusion detection tools assume pairing information will be present. Medium to long reads are desirable to ensure accurate alignment of reads to genes with many paralogs or pseudogenes and also to allow accurate mapping of reads that span across fusion breakpoints that may involve any two points in the genome.
Viral detection [23-25]	Expression of some viruses may be detected and their genome characterized by RNA-seq [23, 100]. In some human tumors, certain viruses may be present either as endogenous elements within the cell or integrated into the genome [25, 101]. In either case it may be possible to detect expression of viral transcripts in RNA-seq libraries generated from these cells. Detection of viral sequences may involve inclusion of certain viral reference sequences (e.g., HPV, HBV, HCV, EBV, etc.) in the reference genome sequence database to which all RNA-seq reads are aligned. Another strategy is to obtain only those reads that do not align to the reference genome sequence for the species being studies and attempt to align these reads to a database of viral sequences. Some strategies further involve *de novo* assembly of these reads into contigs prior to alignment to viral sequence databases.<br>*Data recommendation*: This application requires moderate to high library depth. Detecting sequences that align to viral genomes or distinctive viral k-mers may not require a very deep library if the virus is actively expressing RNAs in the tissue sampled. Identifying fusion sequences involving viruses has many of the same complexities as normal fusion detection and likewise may benefit from longer read lengths.
