# Supplementary material for: Informatics for RNA Sequencing: A Web Resource for Analysis on the Cloud
Source: PLoS Comput Biol. 2015 Aug 6;11(8):e1004393. doi: 10.1371/journal.pcbi.1004393 (PMC4527835; doi:10.1371/journal.pcbi.1004393)
Supplement: S2 Table — All tools used in the online tutorial (www.rnaseq.wiki) are referenced below (tool name in bold) along with alternative tools in each category. Whenever possible, a citation is provided. Links are also provided to help the user evaluate the code and the level of maintenance. Whenever possible, the link goes directly to a source controlled repository such as a git repo. Additional lists of tools can be found here: Alamancos et al. (arXiv), the rna-seqblog, and RNA-seq—Protocols and Algorithms. This table is meant to be comprehensive but not exhaustive. Some RNA-seq analysis topics that are not explicitly covered here include co-regulation (co-expression), disease classification, time series, expression compendium databases, outlier expression, data normalization, and miRNA analysis. (PDF) [file pcbi.1004393.s004.pdf]

S2 Table. Tools for RNA-seq analysis

All tools used in the online tutorial (www.rnaseq.wiki) are referenced below (in bold) along with alternative tools in each category. Where possible a citation is provided. Links are also provided to help the user evaluate the code and the level of maintenance. Where possible the link goes directly to a source controlled repository such as a git repo. Additional lists of tools can be found here: Alamancos et al. (arXiv), Hooper et al. [102], the rna-seqblog, and RNA-seq - Protocols and Algorithms. This table is meant to be comprehensive but not exhaustive. Some RNA-seq analysis applications that are not explicitly covered here include co-regulation (co-expression), disease classification, time series, expression compendium databases, outlier expression, data normalization, and miRNA analysis.

Category	Representative tools
Raw data QC [2, 103]	**FastQC**, HTQC [104], QC3 [105], kPAL [106].
Read trimming [107]	Trimmomatic [58], Skewer [57], **Flexbar [108]**, FASTX.
Alignment (splice aware, for alignment to a reference genome) [59]	**TopHat [84, 109], STAR [110]**, HISAT [111], segemehl [112], GSNAP, MapSplice [113], JAGuaR [114], SpliceMap [115], HMMSplicer [116], TrueSight/UnSplicer [117].
Alignment (non splice aware for alignment to a reference transcriptome) [118, 119]	BowTie [120], Bwa [121].
Post-alignment QC [59]	**FastQC, samtools [56]**, QuaCRS [122], RSeQC [123], RNA-SeQC [124], Picard CollectRnaSeqMetrics, BAMstats, **SAMstat [125]**, BlackOPs [126], seqbias [127].
Gene/transcriptome annotation [128, 129]	Annocript [130], XSAnno [131], GeneMark-ET [132], WImpiBLAST [133], RNASEG [134], TSSAR [135], Vicinal [136], OMIGA [137], CoRAL [138], AfterParty [139], ShortStack [140], CIRI [141].
Small RNA identification and characterization (e.g., miRNAs) [129, 142]	ShortStack [140], CoRAL [138], MTide [143], FlaiMapper [144], miRPlant [145], PROmiRNA [146], omiRas [147], DREAM [148].
Transcript assembly (reference genome guided) [60, 92, 149]	**Cufflinks [8]**, Scripture [150], StringTie [151], bayesembler [152], IsoLasso [153].

Transcript assembly (*de novo*, reference genome free) [60, 149, 154-158]	Trinity [159], Trans-ABySS [9], Oases [160], RSEM [161], DETONATE [11], SEECER (sequencing error correction for assembly) [162], BRANCH [163] (uses partial or related genomics sequences as a guide), EBARDenovo [164], Bridger [165].
Transcript abundance or expression estimation (FPKM/RPKM) [91-94]	**Cufflinks [8]**, eXpress [166], RSEM [161], Sailfish (alignment free) [167], RNA-Skim (alignment free) [168], MITIE [169], ireckon [170], DRUT [171].
Obtaining raw transcript/gene read counts (FPM/RPM) [17]	**HTSeq [172]**, FeatureCounts [173], Rcount [174], maxcounts [175], FIXSEQ (adjusts counts to compensate for overdispersion) [176], Cuffquant.
Differential expression [67-69, 177]	**Cuffdiff [14]**, limma [178], DESeq2 [179], **EdgeR [180]**, Corset (for *de novo* assembled transcriptomes) [181], sSeq [182], BADGE [183], compcodeR [184], metaRNASeq [185], Characteristic Direction [186], NPEBseq [187].
Alternative splicing, alternative expression [94, 102, 188-191]	**Cuffdiff [14]**, DEXSeq [192], ALEXA-seq [3], IUTA [193], FineSplice [194], PennSeq [195], FlipFlop [196], SNPlice [197], spliceR [198], GESS [102], RNASeq-MATS [199], SplicingCompass [200], DiffSplice [201], SigFuge [202], SUPPA [bioRXiv], CLASS [bioRXiv], SplAdder [bioRXiv], SplicePie [203].
Variant (e.g., SNP) and mutation detection [31-33] (germline or somatic) and eQTL/sQTL characterization [204]	GATK (Best Practices Guide) [205], **samtools [56]**, SNVMix [206], SNPlice [197], eSNV-detect [207], RVboost [208], sQTLseekeR [209], eQTL/ASE, [BioRXiv], SNiPloid [210], SNPiR [32], QualitySNPng [211], RNAmapper [212], CRAC [213], RADIA [214].
RNA editing [21, 215-217]	REDItools [218], GIREMI [219], ICEBreaker [220].
Allele specific expression [221-223]	AlleleSeq [19], Allim [224], mamba [225], EMASE, MBASED [226], limma [178].
Viral detection [23-25]	VirusSeq [227], VirusFinder [228], RNA CoMPASS [229].
Fusion detection [26-30]	FusionQ [27], TRUP [230], Dissect [231], Trans-ABySS [9], PRADA (RNA-seq pipeline with a fusion module) [232], Pegasus (used for fusion annotation) [233], FusionCatcher, ChimeraScan [234], TopHat-fusion [235], BreakFusion [236], deFuse [237], FusionHunter [238], EricScript [239], Barnacle [240], bellerophontes [241], Chimera (merge results from multiple fusion algorithms) [29], GFML (format for representing fusion data) [242].

Visualization [243, 244]	SplicingViewer [245], **IGV [62], Sashimi plots [65]**, IGB (splicing visualization protocol) [243], PrimerSeq (Visualize RNA-seq data for primer design) [246], ASTALAVISTA [247], Circos [248], Epiviz [249], RNAbrowse [250], ZENBU [251], RNAseqViewer [252], viRome [253], miRseqViewer [254], Circleator [255], RNASeqBrowser [256].
Integration of DNA-seq and RNA-seq data [257]	Veridical [258], SpliceFinder [259], nFuse [260], RADIA [214].
