# Supplementary material for: Informatics for RNA Sequencing: A Web Resource for Analysis on the Cloud
Source: PLoS Comput Biol. 2015 Aug 6;11(8):e1004393. doi: 10.1371/journal.pcbi.1004393 (PMC4527835; doi:10.1371/journal.pcbi.1004393)
Supplement: S3 Table — The following table summarizes several key concepts relating to sample preparation and library construction that may influence analysis and interpretation of RNA-seq data. Several initiatives are underway to develop standards and best practices that cover many of these concepts. These include the Sequencing Quality Control (SEQC) consortium, the Encyclopedia of DNA Elements (ENCODE) consortium, the Roadmap Epigenomics Mapping Consortium (REMC), and the Beta Cell Biology Consortium (BCBC). (PDF) [file pcbi.1004393.s005.pdf]

**S3 Table. Concepts in sample preparation and library construction that can influence study design, analysis and interpretation**

The following table summarizes several key concepts relating to sample preparation and library construction that may influence analysis and interpretation of RNA-seq data. Several initiatives are underway to develop standards and best practices that cover many of these concepts. These include: the Sequencing Quality Control (SEQC) consortium [2], the Encyclopedia of DNA Elements (ENCODE) consortium, the Roadmap Epigenomics Mapping Consortium (REMC), and the Beta Cell Biology Consortium (BCBC).

| Strategy/concept | Relevance to RNA-seq analysis and data interpretation |
|---|---|
| RNA isolation (RNA integrity and degradation) | RNA is susceptible to degradation, much more so than DNA. RNA degradation can significantly impact library complexity, alignment [261], transcript quantification [262] and other RNA-seq applications. Degradation happens by various mechanisms. Specific sample handling procedures and best practices are commonly employed to maintain intact RNA molecules in solution. For example, RNA isolation is recommended immediately upon obtaining cells. In cases where immediate RNA isolation is not possible, tissues may be stored in preserving agents meant to protect RNA until isolation is possible (e.g., 'RNAlater'). To prevent the degrading activity of endogenous RNAses, RNA isolation involves use of buffers that inhibit the activity of these enzymes. In addition to buffer conditions (e.g., pH), RNA isolation may involve use of specific RNAse inhibitors. Similarly, immediate precipitation and removal of protein (especially RNAses) from the sample reduces the risk of RNA degradation. Use of buffers containing chelating agents inhibits hydrolysis of RNA that can lead to strand cleavage. Best practices such as performing isolation at low temperatures (e.g., on ice) as well as maintaining clean conditions to prevent introduction of exogenous RNAses are commonly employed. At the completion of RNA isolation and prior to sequence library construction, RNA quality is routinely assayed by gel electrophoresis or capillary electrophoresis (e.g., using a Agilent 2100 Bioanalyzer) that provides a qualitatively interpretable 'trace' and single RNA integrity number (RIN). RIN scores vary from 0 to 10. A max score of 10 indicates intact RNA. The lower the score reported, the greater the level of degradation. Many researchers require that RNA isolated from fresh frozen material have a RIN of 6-8 or greater. If RNA is isolated from FFPE archival samples, the RIN will usually be much lower than this. For FFPE materials, alternate strategies may be used to evaluate RNA quality. For example, some researchers choose a 'DV200' cutoff. The DV200 metric describes the percentage of RNA fragments greater than 200 bp in length (refer to this TechNote on TruSeq RNA Access for a more detailed discussion on assessing FFPE RNA quality). Once isolated, RNA is typically stored at -80℃ to inhibit degradation over time. If despite all of these efforts, an RNA sample is degraded, this may result in small fragmented RNA and an RNA-seq library with short |

| | |
|---|---|
| | insert sizes. If fragments are too short, sequencing through the insert may result in a high rate of adapter sequencing. Degraded RNA samples should not be subjected to poly(A) selection to avoid introducing 3' end bias [261]. If an RNA sample is sufficiently degraded, the fragmentation step during library construction may also be skipped. When creating libraries from heavily degraded material, the quality of the resulting library should also be carefully examined. For example, by requiring a minimum concentration (e.g., 5 ng/ul) and that the insert size distribution shows the correct range of fragment sizes. Libraries made from heavily degraded RNA may require extra optimization during the cluster formation step of sequencing. |
| Poly(A) selection versus total RNA versus selective ribosomal RNA reduction (also known as 'ribo-reduction') (**Fig. 4**) | Prior to sequencing, total RNA must be isolated. Total RNA is dominated by ribosomal RNA (rRNA) sequences, comprising 95-98% of RNA molecules. If they are not efficiently removed prior to sequencing, rRNA reads will dominate the data output. Depending on experimental objectives, there are several options for reducing the proportion of rRNAs to allow sequencing of the rest of the transcriptome [49]. Two common strategies are poly(A) selection and selective rRNA reduction. Each has advantages and disadvantages [87]. In poly(A) selection, a solution of oligo(dT) probes is used to capture the poly(A) tail at the 3' end of mature, processed mRNA.<br>In performing poly(A) selection, one is enriching for mature mRNA molecules, leaving behind the pre-processed mRNA as well as other non-coding RNA. In selective rRNA reduction, oligonucleotides homologous to the ribosomal RNAs are used to capture ribosomal RNA that are then removed, enriching for all other RNA species. This procedure will yield sequence reads for non-coding RNA, pre-processed RNA, and other functional RNA molecules like tRNAs. While this data tends to be noisier, it also gives a more broad representation of the transcript classes that make up the transcriptome. |
| Fragmentation (**Fig. 3**) | RNA-seq involves sequencing of cDNA fragments that are usually ~250-450 nucleotides long. The average length of RNA molecules in many species is at least 5-10 times this size. Large RNAs must therefore be fragmented prior to sequencing and the full-length structure of RNAs must be inferred during analysis by assembly of overlapping sequences. Fragmentation is performed directly on the RNA or after conversion to cDNA. RNA fragmentation may be achieved by an enzymatic process (e.g., RNAses), a chemical process (e.g., exposure to metal ions), or a physical process (e.g., exposure to heat or shearing by sonication). cDNA fragmentation may similarly involve an enzymatic process (DNAses), nebulization, or sonication. To obtain a distribution of fragments in a specific size range, fragmentation is often followed by size selection. |
| Size selection (narrow versus | There are two size selection strategies for obtaining cDNA fragments of a size range suitable for RNA sequencing. In the first strategy, a tight |

| | |
|---|---|
| broad size selection versus small-RNA sequencing) (**Fig. 3**) | size range may be selected (by polyacrylamide gel electrophoresis 'PAGE' for example) to produce a distribution with a small variance in size (known as a 'tight' distribution). This allows for efficient cluster formation on a flowcell, leading to a higher data yield from each run. It also allows algorithms downstream to infer more about the structure of RNAs based on any observed deviation from the expected insert size. A small size range reduces the number of possible unique fragments that can be generated from each RNA species and therefore could reduce overall library complexity and sequence content. In the second strategy, only small RNA species are removed using a simple column cleanup that is more amenable to automation in the lab [263]. This leaves a much broader distribution with a long 'tail' of larger RNAs. During analysis, this strategy prevents strong inferences based on calculated insert sizes but the wider diversity of fragments may provide increased sequence complexity and may allow mapping in certain ambiguous regions that might otherwise be difficult to align to. Despite a wide range of sizes, the process of cluster formation and sequencing may be biased towards certain size fragments (likely smaller fragments) and therefore the observed size distribution in sequence reads may be shifted relative to estimates of fragment size obtained prior to sequencing. It should be noted that in both the strategies described above, very small RNAs such as miRNAs are lost. These small RNA species are typically sequenced by an independent small RNA sequencing strategy that specifically targets RNA species in the ~20-150 bp range (or often a further subset of this range). |
| Linear or exponential amplification of low-input samples | To allow for small amounts of input material, certain RNA-seq library construction strategies incorporate an up-front linear or exponential amplification step. Examples of this type of strategy include: 'Smart-seq', 'DP-seq' and 'CEL-seq' [49]. The initial amplification strategy is performed in addition to the exponential PCR amplification that is a routine part of sequence library construction. Any amplification is potentially undesirable as it introduces biases that may mask subtle or even moderate biologically significant differences in RNA expression between conditions [264]. However, in the case of extremely low input samples, some amount of amplification may be required to allow RNA-seq library construction.<br>Linear amplification involves incorporation of an additional adapter sequence containing a promoter sequence that allows a polymerase (often T7 RNA polymerase) to generate copies. The high binding affinity of this enzyme for its promoter sequence is meant to minimize generation of artifactual products that distort expression measurements during analysis; however, this approach has been found to introduce considerable variability at low RNA input levels [264]. During analysis, an additional trimming step is required to remove these promoter sequences. When an initial amplification step is required, additional technical and biological replicates should be considered and greater emphasis placed on data QC during analysis. |

| | |
|---|---|
| Library normalization (also known as cDNA normalization) (**Fig. 4**) | RNAs occur at varying abundances in a cell. These abundances can vary as much as $10^5$-$10^7$, orders of magnitude from the rarest to most abundant transcripts [85, 86]. Since RNA-seq works by random sampling, a typical RNA-seq library is often dominated by reads from the most abundantly expressed genes. With respect to gene expression studies, this is arguably the correct outcome. In studies where measuring the abundance is not as critical as resolving the structures of RNA transcripts, annotating a new genome, or discovering novel RNA fusions, it may be desirable to normalize the library prior to sequencing. Library normalization in this context is any attempt to even out the abundance of transcripts such that the probability of obtaining reads from lowly expressed transcripts and highly expressed transcripts is more balanced. Several RNA-seq library normalization (also known as cDNA normalization) strategies have been proposed. In a completely normalized library, the probability of obtaining reads from all expressed loci would be equal (after correcting for their varying sizes, biases related to GC content, etc.). Duplex-specific normalization (DSN) is one example of a normalization strategy used in RNA-seq library construction [265]. It relies on use of a duplex-specific thermostable nuclease enzyme that preferentially cleaves DNA duplexes and DNA-RNA heteroduplexes. In this strategy, a sequencing fragment library is denatured and partially reannealed before addition of this enzyme. More abundant sequences reanneal more rapidly, and therefore are more heavily degraded by the enzyme, reducing their relative abundance in the library. Note that 'library normalization' described here should not be confused with 'data normalization' that seeks to enable accurate comparisons of expression levels between and within samples by adjusting for systematic biases in the data (i.e. adjusting expression estimates) [95]. Differences in library normalization efficiency between libraries could be one source of bias that might be addressed by data normalization. |
| Exome capture of RNA-seq libraries (and other attempts to recover low quality degraded RNA material such as that obtained from archival FFPE samples) (**Fig. 4**) | One strategy that may be employed to normalize or 'rescue' RNA-seq libraries created from degraded RNA input is to subject them to exome capture (also known as 'cDNA capture'). This approach improves the relative representation of lowly expressed transcripts and concentrates read coverage over the exons targeted by the capture array while reducing the proportion of reads aligning to intronic and intergenic regions. As with all library normalization strategies, this approach could reduce the accuracy of expression estimates. On the other hand, for highly degraded samples (e.g., from FFPE material) it can substantially increase the quality of transcript assemblies compared to uncaptured data [266]. The 'TruSeq RNA Access' kit from Illumina is an example of a commercially available kit that implements the cDNA capture concept. Another method found to be suitable for highly degraded FFPE material is the 'RNAse H' method [49]. Some studies have examined the effects of FFPE input material on results from RNA-seq analysis [267, 268]. |
| Strand specific | RNAs are transcribed by RNA polymerases in a 5' to 3' direction. For |

| | |
|---|---|
| versus unstranded RNA-seq libraries (**Fig. 6**) | the most part, transcription occurs using only a single strand of the double stranded DNA template at any particular locus. However, there are significant portions of the genome where transcription in opposite directions overlaps at the beginning or ends of some genes. Furthermore, transcription of certain genes (e.g., miRNAs) may occur from within the intron of another gene on the opposite strand. In many early RNA-seq library construction strategies, knowledge of which strand had been transcribed was lost. These libraries are referred to as 'un-stranded' libraries. In these libraries we cannot definitively know which strand was being transcribed by RNA polymerase from the genomic DNA template. However, by comparing the position of a read and coverage pattern in that region to known transcript annotations we can often infer the likely direction/strand of transcription. Furthermore, for reads that span across exon-exon junctions, we can compare the observed splice site sequences to that expected for canonical splicing and the strand can often be inferred accurately for these junction spanning reads. Strand specific RNA-seq libraries have the advantage that they maintain the transcription strand info by ligating different RNA adapters on the 5' and 3' ends of each RNA molecule prior to cDNA synthesis [48]. This increases the accuracy of alignment and allows us to independently measure transcription occurring on opposite strands at the same genomic position. Genome browsers capable of visualizing RNA-seq alignments (such as IGV) will often have a setting that allows reads to be colored according to the strand. Read aligners (such as TopHat [84, 109]) and expression estimating tools (such as Cufflinks [8]), and HTSeq Count [172]) also have parameters that should be set to indicate the strandedness of the RNA-seq library (see **Fig. 6** and **S5 Table** for examples). |
| Indexing and pooling of multiple RNA-seq libraries | 'Indexing' in the context of RNA-seq refers to the optional use of a short linker sequence, often a hexamer (or octamer), that is added to the cDNA fragments during library construction prior to sequencing. The index sequence is also known as a 'barcode'. The index may be added to one or both ends of the cDNA fragment during RNA-seq library construction. A distinct index is assigned to each RNA sample to be sequenced. These indexes are often chosen to have a minimum number of sequence differences compared to all other indexes to be used. Once indexed, RNA samples can be mixed, sequenced as a pool and separated during the analysis by a process known as demultiplexing. Accurate demultiplexing relies on exact or near exact matching of the observed index sequence to that expected for each library/sample. Occasional errors will result in some sequences that cannot be demultiplexed and these reads are effectively lost to the analysis unless a custom pre-processing strategy is employed. Once data has been demultiplexed, the index sequence is removed and analysis normally proceeds as it would if no indexing was performed. However, in some cases where short fragments are sequenced and the length of the read exceeds the insert size, it may be possible for index |

| | |
|---|---|
| | sequences to appear in the final read sequence. Such data may benefit from adapter trimming.<br>A multiplexing strategy allows finer control over the amount of data produced for each RNA-seq library. For example, a single lane of RNA-seq data may be divided among 4 or more RNA-seq libraries. With current Illumina protocols, up to 96 samples can be indexed and pooled. The choice to index and pool prior to sequencing is generally driven by the desire to sequence several samples at a depth lower than what is available in a single lane of the instrument (the basic unit of data production). A good rule of thumb for RNA-seq analysis is that if you want only gene expression estimates (similar to what you would get from a microarray experiment) you will need at least ~30-50 million reads of data for each sample [83]. At current data production levels this means that 4-6 samples may be indexed and sequenced within a single lane of Illumina HiSeq 2000 (or equivalent). It should be noted that these estimates assume high quality RNA (e.g., obtained from fresh frozen material) that has been enriched for mRNAs. For FFPE material or total RNA-seq strategies that do not enrich for mRNAs, higher library sequence depth may be required.<br>The sequencing depth, number, and type of replicates are critical to differential gene expression estimates and tools have been created to help design RNA-seq experiments [83, 269]. If the study goals include more detailed analyses such as transcriptome assembly, alternative splicing analysis or single nucleotide variant profiling, the number of reads and replicate libraries required is less well understood. Based on our own data, we recommend up to ~250 million reads per sample (or even more for robust profiling of lowly expressed transcripts).<br>Note: the ‘library indexing’ described here to allow concurrent sequencing of multiple samples in a single lane should not be confused with ‘molecular indexing’ where individual cDNA fragments are labeled to allow each molecule to be tracked from the original sample through sequencing [270]. |
| Use of spike-in controls | Use of a set of synthetic spike-in RNA sequences is suggested as a control during the entire RNA sequencing library preparation process [271]. Introducing the spike-in at the earliest point of RNA sample processing ensures that most of the potential biases introduced during library construction can be accounted for. The spike-ins are used during analysis (alignment and abundance estimation) to evaluate the quality of the sequencing library construction process and account for variability between samples introduced by any number of previously mentioned variables including the sequencing platform used. The spike-in transcript concentrations cover a wide dynamic range to facilitate the calculation of the lower limit of detection, sensitivity and specificity of estimated abundance measurements made during analysis. In addition, the spike-in provides a standard curve that can be used to correct for biases attributed to GC content or positional sequencing bias along the length of transcripts of varying size [271, |

| | |
|---|---|
| | 272]. The 'ERCC RNA Spike-In Control Mixes' (available from Ambion, Catalog Numbers: 4456740, 4456739) are one example of a spike-in reagent. This spike-in consists of 92 transcripts that are present in known concentrations across a wide abundance range (from very few copies to many copies). This range allows us to test the degree to which the RNA-seq assay (including all laboratory and analysis steps) accurately reflects the relative abundance of transcript species within a sample. There are two 'mixes' of these transcripts to allow an assessment of differential expression output between samples by including one of each mix in the two conditions being compared. |
| Sequencing platform and strategy | RNA-seq has been enabled in recent years by the advent of 'next-generation' sequencing, otherwise known as high throughput sequencing, massively parallel sequencing and others names [273-276]. Several companies now offer sequencing platforms that allow dramatic improvements in throughput and reductions in per base cost compared to the previously dominant Sanger sequencing instruments (e.g., the ABI 3730). This increase in data production capacity allows shotgun sequencing to depths sufficient to cover, in a matter of days, the majority of RNA species comprising the transcriptome of any species from which RNA can be obtained. At present there are a few popular sequencing platforms. These include: Illumina (e.g., MiSeq, HiSeq 2000, HiSeq 2500), Ion Torrent (PGM), to a lesser degree Pacific BioSystems, and perhaps in the near future nanopore sequencing platforms such as those produced by Oxford Nanopore. At present the Illumina platform is the most widely used system for RNA-seq. This platform relies on a 'sequence-by-synthesis' approach. Briefly, a solution of DNA molecules are spread across a flowcell surface where individual molecules attach to primers, are amplified by PCR into clusters of identical molecules, and a series of sequential synthesis steps occurs using four types of reversibly terminated nucleotide bases (adenine, cytosine, guanine, and thymine), each fluorescently labeled with a different color fluor. At the end of each step, the terminal base fluoresces under a laser and photographic images of the flowcell are taken. Multiple high resolution photographs are required to cover the surface of the flowcell, which is often divided into 'tiles' and 'lanes'. Image analysis is then used to capture the base identity at each cycle of synthesis for every cluster on the flowcell. The number of cycles determines the read length produced. It is common to produce two sets of reads, one from each end of each molecule captured on the flowcell. If sequenced from one end, the reads are said to be 'single-end' if sequenced from both ends, they are said to be 'paired-end'. In some cases, additional reads are produced if library indexing was used (discussed above). The above procedure is depicted in several illustrative videos online: video 1, video 2. |
