# Supplementary material for: Informatics for RNA Sequencing: A Web Resource for Analysis on the Cloud
Source: PLoS Comput Biol. 2015 Aug 6;11(8):e1004393. doi: 10.1371/journal.pcbi.1004393 (PMC4527835; doi:10.1371/journal.pcbi.1004393)
Supplement: S4 Table — A description of three RNA enrichment strategies is provided, along with their anticipated effects on RNA-seq library construction and data interpretation. For a visual depiction of the concepts discussed here, refer to Fig 4. (PDF) [file pcbi.1004393.s006.pdf]

S4 Table. Description of RNA-seq library enrichment strategies

A description of three RNA enrichment strategies is provided along with their anticipated effects on RNA-seq library construction and data interpretation. For a visual depiction of the concepts discussed here, refer to **Fig. 4**.

	**Enrichment strategy**			
	**Total RNA**	**rRNA reduction**	**PolyA selection**	**cDNA capture**
**General description**	Total RNA is isolated from cells or homogenized tissue. In many species, ribosomal RNA (rRNA) comprises as much as ~95-98% of all RNA molecules. For this reason, total RNA is rarely used for RNA-seq without first conducting an enrichment of some kind.	A strategy that attempts to capture rRNAs by hybridization to specific oligonucleotides. While rRNAs are immobilized, all other RNA molecules are washed through and used as input for RNA-seq library construction. The enrichment for RNAs of interest in indirect in this strategy.	A strategy that attempts to directly capture RNAs containing a polyA tail by hybridization to specific oligonucleotides. While polyadenylated RNAs are immobilized, all other RNA molecules are washed away. After washing, the polyadenylated RNAs are eluted and used as input for RNA-seq library construction.	A strategy that attempts to directly capture RNAs homologous to known exon sequences, for example by using an exome capture reagent. While this set of RNAs are immobilized, all other RNA molecules are washed away. After washing, the captured sequences are eluted and used as input for RNA-seq library construction.
**Description of oligo/capture molecules**	No capture technique is used.	Sequences complementary to the rRNA transcripts of the species of interest. In human, rRNAs are divided into: 5S (120 bases in length), 5.8S (160 bases), 18S (~1.9 kb), and 28S (~5 kb) RNAs. Oligonucleotides in rRNA reduction kits attempt to target each of these rRNA sequences.	Oligonucleotides consisting of a series of thymine nucleotides complementary to the polyA tail of mature mRNA molecules. These sequences, often of 18-20 bases in length are referred to as oligo(dT)s.	cDNA capture sets can vary considerably, but consist of a broad selection of oligonucleotides with sequences complementary to known transcript sequences. In the case of an ‘exome’, designed oligos may cover a significant portion of all known exons for the species of interest.
**Transcriptome representation**	Most broad. Close to an unbiased, complete representation.	Broad representation.	Focused transcriptome representation (polyA RNAs only).	Varies. Most focused transcriptome representation (targeted sequences only).
**Effect on rRNAs**	Total RNA contains large amounts of rRNA along with all other classes of RNAs	Low rRNAs. rRNAs may be reduced by 60-90% or more.	Very low rRNAs. rRNAs may be reduced by 80-90% or more.	Very low rRNAs. rRNAs may be reduced by 80-90% or more.

**Effect on abundant RNAs (other than rRNAs)**	While rRNAs dominate in total RNA, the remaining RNAs of other classes occur at widely different proportions, varying by at least 5-6 orders of magnitude from the least to most abundant transcript.	rRNA reduction should not effect the relative abundance of RNAs other than the rRNAs.	PolyA selection will result in an enrichment of polyA RNA molecules at the expense of rRNAs and all non-polyadenylated RNA (including many non-coding RNAs). However, within the polyA RNAs, relative abundance differences should remain unchanged.	cDNA capture will result in an enrichment of all RNA sequences targeted by the capture reagent at the expense of rRNA and all other RNAs that are not targeted. The difference between the most highly and lowly expressed transcripts that are targeted by the capture reagent may also be reduced. This 'compression' of dynamic range occurs because highly expressed genes may 'saturate' the corresponding capture probes.
**Effect on rare RNAs**	Rare RNAs would be extremely difficult to observe by RNA-seq of total RNA because it works by random sampling of fragments. Rare RNA molecules would have a very low probability of being sequenced. Almost all sequenced fragments would align to rRNAs.	rRNA reduction focuses the total pool of RNA-seq reads onto all RNA classes other than rRNA. However, among the very diverse pool remaining, rare transcripts are still difficult to observe by random read sampling.	PolyA selection focuses RNA-seq reads onto an even narrower subset of the transcriptome than rRNA reduction. This improves the ability to detect rare polyA transcripts but the most highly expressed transcripts will still dominate.	cDNA capture is the most focused strategy described here and most reads will correspond to a target of interest. Furthermore the most abundant transcripts targeted by the capture are reduced, increasing the ability to sequence very rare transcripts.
**Effect on genomic DNA (gDNA) contamination**	Unaffected. Any gDNA contamination remaining after RNA isolation (and likely DNAse treatment) would be sequenced.	rRNA reduction is not expected to substantially affect the overall level of gDNA contamination.	Since gDNA sequences are not polyadenylated, their relative presence should for the most part be reduced following selection for polyA transcripts. However, regions of the genome with polyA stretches may also be inadvertently captured by oligo(dT) probes.	Overall, gDNA contamination will be reduced by cDNA capture except for gDNA fragments that substantially overlap the targeted sequences of the capture reagent. Signal from intergenic and intronic reads should be substantially reduced.

**Effect on unprocessed RNA (also known as hetero-nuclear RNA, hnRNA)**	Unaffected. Any unprocessed RNA contamination remaining after RNA isolation would be sequenced.	rRNA reduction is not expected to substantially affect the overall level of unprocessed RNA contamination.	Unprocessed RNA should be significantly depleted. Since polyA tail addition occurs near the end of RNA transcription when the transcript emerges from an RNA polymerase complex, performing a polyA selection will tend to enrich for mature mRNAs that have been completely processed.	cDNA capture probes generally target the exons of known transcripts directly. For this reason, intronic RNA-seq reads corresponding to unprocessed RNA should be reduced. Signal from unprocessed RNA may still be considerable near the edges of targeted exons.
