# Supplementary material for: Informatics for RNA Sequencing: A Web Resource for Analysis on the Cloud
Source: PLoS Comput Biol. 2015 Aug 6;11(8):e1004393. doi: 10.1371/journal.pcbi.1004393 (PMC4527835; doi:10.1371/journal.pcbi.1004393)
Supplement: S5 Table — This table provides further explanation of IGV’s read orientation codes for RNA-seq data viewed in the browser. Also provided are recommended software settings for three additional tools involved in common RNA-seq analysis workflows: TopHat, HTSeq, and Picard. Each of these explanations/settings is provided for several commonly used RNA-seq library construction kits that produce either stranded or unstranded data. (PDF) [file pcbi.1004393.s007.pdf]

S5 Table. Strand related settings for RNA-seq tools that must be adjusted to account for library construction strategy

This table provides further explanation of IGV's read orientation codes for RNA-seq data viewed in the browser. Also provided are recommended software settings for three additional tools involved in common RNA-seq analysis workflows: TopHat [84, 109], HTSeq [172], and Picard. Each of these explanations/settings is provided for several commonly used RNA-seq library construction kits that produce either stranded or unstranded data.

Library Kit	Stranded	5' to 3' IGV	TopHat (--library-type parameter)	HTSeq (--stranded/-s)	Picard (STRAND_SPECIFICITY option of CollectRnaSeqMetrics)
TruSeq Strand Specific Total RNA	Yes	F2R1	fr-firststrand	reverse	SECOND_READ_TRANSCRIPTION_STRAND
NuGEN Encore	Yes	F1R2	fr-secondstrand	yes	FIRST_READ_TRANSCRIPTION_STRAND
NuGEN OvationV2	No	F2R1 or F1R2	fr-unstranded	no	NONE

To identify which '--library-type' setting to use with TopHat [84, 109], Illumina specifically documents the types in the 'RNA Sequencing Analysis with TopHat' Booklet. For the TruSeq RNA Sample Prep Kit, the appropriate library type is 'fr-unstranded'. For TruSeq stranded sample prep kits, the library type is specified as 'fr-firststrand'. These post are also very informative: How to tell which library type to use (fr-firststrand or fr-secondstrand)? and How to determine if a library Is strand-specific. Another suggestion is to view aligned reads in IGV and determine the read orientation by one of two methods. First, you can have IGV color alignments according to strand using the 'Color alignments' by 'First-of-pair strand' setting. Second, to get more detailed information you can hover your cursor over a read aligned to an exon. 'F2 R1' means the second read in the pair aligns to the forward strand and the first read in the pair aligns to the reverse strand. For a positive DNA strand transcript (5' to 3') this would denote a fr-firststrand setting in TopHat, i.e. "the right-most end of the fragment (in transcript coordinates) is the first sequenced". For a negative DNA strand transcript (3' to 5') this would denote a fr-secondstrand setting in TopHat. 'F1 R2' means the first read in the pair aligns to the forward strand and the second read in the pair aligns to the reverse strand. See above for the complete definitions, but it's simply the inverse for 'F1 R2' mapping. Anything other than FR orientation is not covered here and discussion with the individual responsible for library creation would be required. Typically 'RF' orientation is reserved for large-insert mate-pair libraries. Other orientations like 'FF' and 'RR' seem impossible with Illumina sequence

technology and suggest structural variation between the sample and reference. Additional details are provided in the TopHat manual.

For HTSeq, the htseq-count manual indicates that for the '--stranded' option, 'stranded=no' means that a read is considered overlapping with a feature regardless of whether it is mapped to the same or the opposite strand as the feature. For 'stranded=yes' and single-end reads, the read has to be mapped to the same strand as the feature. For paired-end reads, the first read has to be on the same strand and the second read on the opposite strand. For 'stranded=reverse', these rules are reversed.

For the 'CollectRnaSeqMetrics' sub-command of Picard, the Picard manual indicates that one should use 'FIRST_READ_TRANSCRIPTION_STRAND' if the reads are expected to be on the transcription strand.
