# Supplementary material for: Informatics for RNA Sequencing: A Web Resource for Analysis on the Cloud
Source: PLoS Comput Biol. 2015 Aug 6;11(8):e1004393. doi: 10.1371/journal.pcbi.1004393 (PMC4527835; doi:10.1371/journal.pcbi.1004393)
Supplement: S6 Table — The following table describes several file formats used in most RNA-seq analysis workflows as well as several files specific to the expression analysis tools used by the online tutorials that accompany this article (at www.rnaseq.wiki). (PDF) [file pcbi.1004393.s008.pdf]

**S6 Table. Standard file formats and tool-specific files used in RNA-seq analysis**

The following table describes several file formats used in most RNA-seq analysis workflows as well as several files specific to the expression analysis tools used by the online tutorials that accompany this article (at www.rnaseq.wiki).

| File type | Description |
|---|---|
| FASTA [54] | http://www.ncbi.nlm.nih.gov/BLAST/blastcgihelp.shtml<br><br>FASTA files are used to store sequences of DNA with a header that describes its source. It is the preferred format to represent the reference genome sequence needed by analysis algorithms like BLAST, BLAT, bwa, bowtie and TopHat. |
| GTF | http://www.ensembl.org/info/website/upload/gff.html<br><br>GTF (a constrained version of GFF), or gene transfer format, is a format that describes DNA, RNA, or protein sequences with their chromosome location and basic structural and functional annotations. |
| FASTQ [55] | http://maq.sourceforge.net/fastq.shtml<br><br>FASTQ format is a next generation sequencing specific format for storing read sequence data. It includes a read quality score along with FASTA-like sequence information. This format is used to describe each RNA-seq read individually and is an accepted input to most sequence aligners. |
| SAM/BAM [56] | http://samtools.github.io/hts-specs/SAMv1.pdf<br><br>SAM (Sequence Alignment Map) is a flexible sequence alignment format used to describe the alignment of sequence reads to a reference genome sequence. BAM is a binary, compressed version of the SAM file used for more efficient storage and access. |
| BED | http://www.ensembl.org/info/website/upload/bed.html<br><br>BED (Browser Extensible Data) is a file format that is used to store location-annotation genome coordinate pairs to be displayed in a genome browser. |
| junctions.bed [84, 109] | https://www.biostars.org/p/16653/<br><br>This file format is somewhat specific to the TopHat tool though it follows the general convention of BED files as described above. The junctions.bed file is produced by running TopHat on a set of read sequences. It contains exon-exon junction (and exon boundary) information and counts for all reads spanning two exons across an intron. |

| | |
|---|---|
| Cufflinks output files [8] | https://www.biostars.org/p/16574/<br>http://cole-trapnell-lab.github.io/cufflinks/cufflinks/index.html#cufflinks-output-files<br><br>Cufflinks produces two main output files. (1) A transcripts.gtf file, an annotated file of the transcript sequences/structures predicted by Cufflinks by examining RNA-seq read alignments. (2) fpkm_tracking files, that are used to summarize expression values at both the gene and the transcript level. The format of both of these files is somewhat specific to the Cufflinks tool, though the transcripts.gtf file follows the general convention of GTF files as described above. |
| HTSeq output files [172] | http://seqanswers.com/forums/showthread.php?t=4805<br>http://www-huber.embl.de/users/anders/HTSeq/doc/count.html<br><br>HTSeq produces a simple tab delimited output file, specific to the HTSeq tool, with raw read counts summarized to the level of specific genome features. Usually the count represents reads that overlap any of the exons of a gene and one value is reported for each gene. |
