# Supplementary material for: Informatics for RNA Sequencing: A Web Resource for Analysis on the Cloud
Source: PLoS Comput Biol. 2015 Aug 6;11(8):e1004393. doi: 10.1371/journal.pcbi.1004393 (PMC4527835; doi:10.1371/journal.pcbi.1004393)
Supplement: S7 Table — The following table summarizes a list of commonly asked questions relating to RNA-seq analysis, with links to BioStar posts in which these questions have been addressed by the community. (PDF) [file pcbi.1004393.s009.pdf]

S7 Table. Common RNA-seq analysis questions and their answers.
The following table summarizes a list of commonly asked questions relating to RNA-seq analysis with links to BioStar [90] posts where these questions have been addressed by the community.

**Question**	**BioStar posts with answer**
Where can I find a list of RNA-seq analysis review papers?	https://www.biostars.org/p/52152/<br><br>In addition to the biostars link provided we have created a resources page that contains many useful papers and other RNA-seq references at: www.rnaseq.wiki.
General discussion of RNA-seq analysis pipelines and best practices	https://www.biostars.org/p/6615/<br><br>In addition to the BioStar URL, we provide additional references relating to analysis pipelines and best practices in **S8 Table**.
Should I include biological replicates in my RNA-seq experimental design? If so, how many?	http://www.biostars.org/p/1161/<br>http://www.biostars.org/p/68885/<br><br>Yes. RNA-seq can be used to quantify transcript levels from a sample. In order to perform useful statistics, one sample is insufficient. Replicates must be used to appropriately power such statistics. The RNA-seq method is an impressive advancement with many applications for studying RNA biology but it does not eliminate biological variability. If the input samples are heavily degraded or have very low input amounts it may also be advisable to include certain types of technical replicates (e.g., making multiple libraries from each sample). Some studies have shown that for differential expression analysis, use of additional biological replicates holds greater value than greater depth once you achieve 10M reads per samples [277]
How much RNA-seq data should I generate? How much total coverage do I need?	https://www.biostars.org/p/65501/<br><br>The question of how much coverage is necessary for an experiment is very difficult to answer and depends on experimental goals [278]. A common target used at our center is that at least 10,000 transcripts have at least >20x coverage over at least 50% of their known exon-exon junctions. This is usually obtained by a 200-300 million read run of 1-2 lanes of Illumina HiSeq data (40-100 Gb). The ENCODE consortium and other large-scale sequencing initiatives have also published guidelines on this question (**S3 Table**).

	A more precise answer to this question depends on a number of factors, but the most important of these is the analytical question being asked of the data [278]. For example, the experiment may call for gene expression estimates, *de novo* transcriptome assembly, alternative expression analysis, or fusion detection. Published reports have argued that as little as 10 million reads are sufficient for gene expression estimation for each sample [277]. While there are clear statistical benefits to additional samples at the expense of deeper data on each sample [53, 67], these estimates often assume that gene expression estimates are the only desired output of an RNA-seq experiment. Fusion detection, alternative expression analysis and other analysis strategies place higher demands on library depth for each sample. The optimal target sequence depth may also depend on the tissue type being profiled, method of RNA isolation, quality of input RNA, library construction method, and other experimental design factors (**S3 Table**). Furthermore, sequencing parameters such as read length or choice of paired versus unpaired read types influence read alignment efficiency and therefore may influence the total amount of read data needed. Given the number of factors involved, there is no single right answer as to the amount of RNA-seq data needed. One strategy for setting this experimental design parameter is to base the decision on comparison to existing publications with similar goals. A more reliable approach is to determine analysis goals, identify metrics that measure the desired output (genes detected, exon-exon junctions resolved, etc.) and conduct a pilot experiment where a small subset of your libraries is sequenced deeply. The resulting data can then be analyzed, saturation curves produced and the amount of data needed can be determined by a return on investment analysis [269].
How do I assess the quality of an RNA-seq library? What tools are available?	https://www.biostars.org/p/103090/<br><br>Initially, RNA-seq quality control is performed in the lab by evaluating the RIN value of a starting RNA sample (e.g. RIN > 6), insert size distribution of the library (e.g. within target size and not too many fragments below 200 bp) as well as requiring a minimum library concentration before sequencing (e.g. >5 ng/ul) (**Fig. 3**, **S3 Table**, and **S1 Data)**. Sequence instrument quality is usually included in the software provided by the instrument vendor. Common measures of instrument QC are the base quality, nucleotide distributions across the run over time and spatial distributions of base quality and nucleotides within the sequencing media (e.g., flowcell). After sequencing of the library, generic quality control programs for

	sequencing data include: samtools, FastQC, BAMstats and SAMstat (see *Raw data QC* in **S2 Table**). Pre-alignment QC involves an assessment of the nucleotides sequenced and the over representation or bias of nucleotides within the read. Standard DNA quality values differ for RNA-seq in this regard. For example, the GC content of the transcriptome will have a different distribution than the whole genome. Depending on the transcripts expressed in the sample, the sequenced GC content can also vary by sample. After alignment, the simplest RNA-seq QC is an assessment of unique and multi-mapped reads. TopHat and most RNA-seq aligners produce summary metrics about the output alignments in addition to the aforementioned tools. Additional important RNA-seq specific QC metrics include the amount of sequence generated that aligns to coding regions, ribosomal genes and splice junctions. Picard's CollectRNASeqMetrics tool is very useful for RNA-seq QC. The junctions.bed file from TopHat is also useful for determining the sequence coverage of known and/or novel splice junctions. Many additional metrics for evaluating RNA-seq data quality have been developed [103], also see *Post-alignment QC* in **S2 Table**.
Should I trim RNA-seq reads. What trimming tool should I use?	https://www.biostars.org/p/84305/<br><br>Read trimming may be advisable in certain circumstances depending on the results of QC analysis of the data. For example, if there is a considerable drop in base quality near the 5' end of the reads, then quality trimming can be used to remove bases with an increased probability of containing errors. If too many errors are present at the ends of reads this may reduce overall alignment rates. If the RNA-seq library contains cDNA inserts that are shorter than the target read length, sequencing may run into the sequencing adapters used by the sequencing platform. These sequences may prevent reads from mapping to the reference genome. These reads can be fixed by adapter trimming with the known sequence of each sequencing adapter. Finally, if the RNA-seq library construction procedure involved an amplification step that required addition of an additional adapter sequence (e.g., T7 promoter or SPIA adapter) then additional adapter trimming may be advisable. Several read trimming tools are available for next generation sequence data including: Skewer [57] and Trimmomatic [58].
What is 3' end bias and how might it complicate interpretation of expression estimates?	http://seqanswers.com/forums/showthread.php?t=9839<br>https://www.biostars.org/p/102812/<br><br>It is difficult to produce a library with perfectly uniform coverage of RNA-seq reads across the entire length of

	transcripts. For example, base positions at the extreme ends of transcripts tend to be slightly underrepresented at both the 5' and 3' end because there are less cDNA fragments that can be generated from the ends that would cover these positions than in the center of a transcript [279]. The term 3' end bias in the context of RNA-seq refers to an overrepresentation of read sequences derived from the 3' end of transcript. This bias towards sequencing the 3' ends of transcripts can be introduced by certain library construction strategies. In particular if the starting RNA is degraded (or becomes degraded during sample preparation) and the sample is then subjected to polyA enrichment, this will introduce 3' end bias [49]. If the level of RNA degradation is high, the resulting sequence can be almost entirely focused on the 200-400 bases at the 3' end of each transcript. Tools such as Picard can produce visualizations and specific metrics to assess the degree of end bias in an RNA-seq data set and specific methods have been proposed to correct for positional bias in RNA-seq expression estimation [279].
Where do I obtain reference genome sequences (FASTA files) for my species of interest?	https://www.biostars.org/p/1796/<br>https://www.biostars.org/p/103359/<br><br>Reference genome sequences are generally obtained as a set of FASTA sequences representing the results of a genome sequencing and assembly initiative. The assembly consists of multiple contig sequences that each represent an entire chromosome or pieces of chromosomes depending on the degree of completion of the genome assembly. There will often be multiple versions of the genome assembly that represent ongoing improvements (e.g., hg17, hg18, hg19 for *homo sapiens*). Many species have a dedicated reference genome consortium and may operate an independent data portal where these sequences can be downloaded. Furthermore UCSC, Ensembl, and NCBI each act as centralized portals where reference genome sequences can be obtained for multiple species. Finally, the iGenomes project is hosted by Illumina and attempts to provide reference sequences that have been pre-indexed and organized for certain RNA-seq analysis workflows.
Where can I obtain reference transcript sequences (GTF files) for my species of interest?	https://www.biostars.org/p/108359/<br><br>Transcriptome databases contain predicted and/or experimentally validated RNA transcript sequences that have been annotated against the reference genome sequence to resolve exon/intron boundaries. Additional functional annotations may also be available for each transcript sequence or gene locus. Transcript sequences are often

	made available as a FASTA file and annotations of those transcripts against the reference genome (including exon coordinates on the reference genome) will be provided as a GTF or GFF file (**S6 Table**). The same organizations described in the previous question that make the reference genome sequences available also make these transcriptome databases available for download.
Which aligners are optimized for RNA-seq and which should I use?	https://www.biostars.org/p/60478/<br><br>TopHat [84, 109] is a popular choice for RNA-seq alignment. STAR [110] is an alternative that produces similar alignments more quickly. If reads are being aligned against a reference genome sequence, the aligner used should be a gapped aligner that is aware of splicing patterns for the species being sequenced. If reads are being aligned directly to a database of transcript sequences, a faster aligner that is not splice aware may be used. Many alternatives to TopHat are available [59], each with their own benefits and shortcomings. A large list of such aligners is maintained at the EBI HTS aligner list (rna-seq aligners are indicated in red) [118].<br><br>The optimal alignment strategy depends on read length and the availability or choice of reference sequences that the reads are being aligned to. If read lengths are sufficiently long (>75 bp) and they are being aligned to a reference genome sequence, a gapped or 'splice aware' aligner such as TopHat [84, 109], STAR [110], MapSplice [113], GSNAP [280], HISAT or others should be used for a eukaryotic species where exon sequences may be separated by large introns that must be resolved during alignment. If read lengths are < 50 bp it may be advisable to use an ungapped aligner like BWA or Bowtie to align reads to a reference genome combined with an exon-exon junction database [3]. In this strategy, the junction database should be tailored to read length. In the absence of a reference genome sequence, RNA-seq reads can be aligned directly to a database of transcript sequences using an ungapped aligner. In the absence of a reference genome sequence or reference transcriptome database, *de novo* transcriptome assembly may be attempted with tools such as Trans-ABySS [9] or Trinity [10]. For some species such as human, the reference genome and transcriptome resources available are of high quality, having been created by extensive efforts involving gold standard sequencing and analysis techniques. Use of a reference genome and transcriptome to guide and inform the analysis is highly recommended where possible. *De novo* assembly and de convolution of alternative isoforms are difficult problems compared to alignment of reads to a high

	quality reference genome sequence and comparison to a database of known transcripts [11]. *De novo* transcriptome assembly may be used to compliment transcript discovery workflows that are guided by existing reference genome and transcriptome sequences. If these resources do not exist for a particular species, their creation should be considered a high priority.
Is one alignment strategy sufficient for all downstream analysis needs?	Unfortunately, some tools for certain RNA-seq analysis applications have been carefully tuned to expect certain very specific alignment strategies. For example, one transcript abundance tool might expect alignments performed against a reference genome sequence while another might expect alignments performed against a database of transcript sequences. Fusion detection algorithms may rely on alignments that report many alternative alignments. Mutation calling tools might expect a BAM with duplicates marked while most other applications will not be affected by or require duplicate marking. Some RNA-seq aligners do not report small insertions or deletions very well and this will interfere with detecting variants of this type. Some aligners may not report alignments that span across two chromosomes, and this will also prevent detection of fusions. For these reasons and more one should consider carefully the alignment requirements of each analysis application and accept the reality that aligning the same data more than once by different methods might be a necessity in a comprehensive analysis pipeline.
Should I allow multiple alignments for each read?	The answer to this question depends on the application. In DNA analysis it is common to use an alignment strategy that randomly selects one alignment from a series of equally good alignments. In RNA-seq analysis this is less common. When aligning RNA-seq reads against a transcript sequence database, multiple equally good alignments will be expected for genes with several isoforms that share common sequences. Some transcript abundance estimation tools (e.g., Cufflinks [8]) specifically expect to use multiple mappings to a transcriptome or genome sequence in their estimations. Correctly representing the uncertainty of mapping for reads that correspond to multiple isoforms or regions of the genome has been found to increase the accuracy of transcriptome abundance estimation [281, 282]. In other words, allowing more multiple alignments is desirable in this context, though it will increase the size of RNA-seq BAM files. Similarly, in gene fusion discovery, allowing a larger number of alignments for each read can improve the ability of the fusion detector to correctly identify false positive fusions. One use case where one might choose to ignore

	multi-mapped reads is when performing mutation discovery with RNA-seq data. In this application, it might be best to align reads to the genome with an accurate gapped aligner and assign multi-mapped reads a mapping quality of 0 so that they can be easily ignored by variant callers interrogating the BAM file.
Why are there so many RNA-seq alignments within intronic regions?	https://www.biostars.org/p/42890/<br><br>RNA-seq alignments within intron regions can occur for various reasons [283]. First, while it is typical to perform DNAse treatment of RNA samples prior to library construction, these treatments are not complete and some intronic reads may represent genomic DNA that was not successfully removed or degraded. Second, RNA samples will typically contain a mixture of nuclear and cytoplasmic RNA. RNA from the nucleus may be incompletely processed heteronuclear RNA (hnRNA). hnRNA may contain introns that have not yet been spliced out. Third, random transcription events can happen anywhere, including within introns. Fourth, splicing errors or biologically significant alternative splicing may result in isoforms with retained introns. Fifth, the read may be misaligned to the intron. Sixth, if the RNA-seq library is unstranded, such reads might actually correspond to a gene being transcribed on the opposite strand that happens to reside within the intron of another gene. RNA-seq libraries that involve polyA selection will generally enrich for mature mRNA sequences that have been completely processed. This will lead to reduced noise levels within the introns. Another strategy to reduce intron reads might be to perform RNA isolation in a way that enriches for the cytoplasmic compartment or that selects for RNAs being actively translated by a ribosomal complex. Unfortunately, these strategies tend to lead to RNA degradation compared to conventional RNA isolation procedures.
What is a duplicate read?	http://seqanswers.com/forums/showthread.php?t=6854<br>http://sourceforge.net/p/picard/wiki/Main_Page/<br>https://www.biostars.org/p/107402/<br><br>Duplicate reads are two or more reads that are assumed to be derived from the same nucleotide fragment and therefore do not represent independent transcriptome information from the sample being sequenced. Duplicate reads are identified by algorithms that examine position sorted BAM files. Typically, for paired-end read data (single-end data is also handled) these algorithms find the 5' coordinates and mapping orientations of each read pair while taking into

	account all clipping that has taking place as well as any gaps in the alignment. All read pairs sharing identical 5' coordinates and orientations are marked as duplicates except the "best/first" pair. Two commonly used tools for duplicate marking/removal are Picard 'MarkDuplicates' and samtools 'rmdup'. Note: This question/answer refers to PCR duplicates, 'optical duplicates' are a distinct concept.
Should I remove duplicates from RNA-seq libraries?	https://www.biostars.org/p/14283/<br><br>Generally no, but the decision to remove duplicates could be made on a case-by-case basis for your dataset. Unlike in DNA sequencing studies, duplicate reads in an RNA-seq sample are much more likely to be real identical fragments of small RNA transcripts with high expression. Removing these would bias the expression distribution of your sample and is not recommended [83]. However, if quantification of expressed transcripts is not the aim of the study, then removing duplicates can reduce memory usage and computing time for other analyses.<br><br>Duplicate read removal is a standard practice in WGS and exome sequencing pipelines and involves the identification and marking of read alignments that are deemed identical to each other. Duplicates are typically identified as those read pairs that share identical outer alignment coordinates for both reads of a pair (see previous answer for more details). These identically mapped reads are assumed to be artifacts of PCR amplification derived from the same DNA fragment because the probability of sequencing an identical fragment of DNA from genomic DNA by chance is low. While this assumption holds for DNA (from species with large genomes) it does not hold for RNA. There is a concern that duplicates may correspond to biased PCR amplification of particular fragments, however, for highly expressed or short genes, duplicates are expected even if there is no amplification bias. Removing them will reduce the dynamic range of expression estimates. Generally duplicates should therefore not be removed in RNA-seq analysis. However, in some situations (such as mutation calling) one might decide to remove them.
What does 'Fragments Per Kilobase Of Exon Per Million Fragments Mapped' (FPKM) mean?	https://www.biostars.org/p/68126/<br><br>FPKM is an expression estimate that attempts to normalize for differences in library sequence depth between samples and differences in gene size between genes. FPKM is a similar metric to Reads Per Kilobase of transcript per Million (RPKM). However, FPKM values use the count of cDNA fragments, not reads. Various sequencing platforms can

	generate single or paired end reads, introducing ambiguity in the mapping from reads to fragments. FPKM values attempt to resolve this ambiguity by using the fragment of cDNA as the smallest unit. Cufflinks is an example of a tool that generates FPKM values for genes and transcripts/isoforms [8].
How are individual reads assigned to specific transcripts/isoforms when calculating FPKM?	https://www.biostars.org/p/16649/<br><br>The problem of assigning individual reads to specific isoforms or transcripts is a challenging one. Current popular solutions take many inferences into account in determining isoform structures with read counts. Some of the ambiguity in this problem can be resolved by local differences between isoform structures that can be mapped uniquely, but caution should be taken before interpreting the FPKM values for specific isoforms with large, complex splicing patterns.
How do I find novel splicing events/transcripts? What tools are available for alternative splicing detection from RNA-seq data?	https://www.biostars.org/p/68966/<br>https://www.biostars.org/p/65617/<br><br>This problem is still being actively addressed. Separating the problem into subtasks can be useful. Breaking up the alignment, assembly and transcript calling and quantification may lead to a cleaner solution, and many tools are available for these tasks at the links above.
How do I obtain read counts for those reads that span across exon-exon junctions?	https://www.biostars.org/p/73832/<br><br>If alignments were produced by TopHat [84, 109], the exon-exon junctions and read counts supporting each unique junction will be provided in a 'junctions.bed' file in the TopHat output directory. More generally, one could identify alignments in an RNA-seq BAM file that contained CIGAR strings with 'N' operators that indicated skipped regions from their reference. A subset of these skipped regions will correspond to introns. These can be identified by examining the edges of the skipped region and using knowledge of splicing patterns in the sequenced species to determine whether it represents a likely intron splicing event.
How do I visualize alternative splicing events in RNA-seq data?	https://www.biostars.org/p/8979/<br><br>Alternative splicing events are often visualized in genome browsers such as IGV [62] by observing the splice junction spanning reads in the read alignment track, or by loading a 'junctions.bed' file that summarizes read counts supporting exon-exon junctions, or by using a genome browser plugin such as the 'Sashimi plot' module [65] in IGV. Detailed

	protocols for visualizing alternative splicing in the genome browser IGB [64] have also been developed [58]. Additional options are discussed in the biostars post linked above.
How can I generate a custom isoform structure diagram (exon/intron boundaries)?	https://www.biostars.org/p/17841/<br><br>This is possible in R, Perl, or Bioperl graphics utilities. Online tools exist as well. GenomeGraphs and the ExonIntron tool are two such applications. The cummerbund bioconductor package can also create such visualizations for isoforms predicted by Cufflinks in the Tuxedo suite of tools.
How do I detect gene fusions in RNA-seq data? What tools are available?	https://www.biostars.org/p/45986/<br><br>Gene fusions are mostly analyzed in the context of cancer transcriptomes, where several prominent oncogenic fusion proteins are well described (e.g., *BCR-ABL*). Gene fusions are detected by identifying RNA-seq reads that indicate that portions of two genes (gene A - gene B) at physically separated genomic loci are expressed as a single unit. Since transcription normally occurs as a linear event in the 5' to 3' direction along a single continuous DNA molecule, such fusions observed at the RNA level may imply the presence of a structural variation (e.g., interchromosomal translocation) at the DNA level. RNA-seq reads that support a fusion are typically of two categories: spanning and encompassing. A spanning read is one where a single read sequence matches for part of its length to geneA and matches geneB for the remainder. The edges of these alignments to geneA and geneB often correspond to the edge of known exons. An encompassing read is one where read 1 of a read pair matches geneA and read 2 of the same read pair matches geneB. The details for many published fusion detection tools are available at the URL above.
Where can I obtain publicly available RNA-seq datasets?	http://www.ncbi.nlm.nih.gov/geo/<br>https://www.biostars.org/p/46059/<br>https://www.biostars.org/p/52866/<br>http://seqanswers.com/forums/showthread.php?t=20469<br><br>The largest repository of publicly available RNA-seq datasets is the Gene Expression Omnibus hosted by NCBI NLM. Other sources are discussed in the links provided above.
Where can I find a "gold standard RNA-seq data set" for differential expression analysis?	https://www.biostars.org/p/78229/<br><br>The experimental data reported in the ALEXA-seq publication is likely still the most in-depth validated data set publicly available [3]. The GEO accession for this data is GSE23776.

	This data contains ~200 differentially expressed exons validated by qPCR, and another ~200 alternative splicing structures validated by RT-PCR and Sanger sequencing. An additional data set compared various RNA-seq protocols to qPCR data for 40 genes [264]. Projects such as BioXpress [284] and GTEX [285] attempt to summarize existing publicly available RNA-seq data.
How do I integrate RNA-seq expression and gene regulation analyses?	https://www.biostars.org/p/11695/ <br>While limited tools currently exist, there is great potential to combine whole genome or exome data generated by sequencing DNA with RNA-seq data generated by sequencing RNA from the same samples. This will allow an unprecedented ability to examine the sequence relationship between common polymorphisms and rare mutations in the DNA with expression levels and splicing patterns in the RNA.
Should I perform data normalization of gene/transcript expression estimates? How?	https://www.biostars.org/p/88751/ <br>Normalization of gene or transcript expression estimates is performed for a number of reasons. Most commonly the aims are to ensure that (1) estimates are comparable within samples (e.g., comparing expression of one gene to another); (2) estimates are comparable between samples (e.g., comparing expression of a gene across experimental conditions) and (3) estimates are in scales or distributions that are convenient for interpretation and visualization. It is almost always necessary to perform at least some kind of normalization. The simplest methods such as transcripts per million (TPM) or fragments per kilobase of exon per million reads mapped (FPKM) attempt to control for differences in library depth and/or feature (e.g., gene) size with simple transformations. Note that methods such as TPM only control for differences in feature size and thus can be used to compare within samples, but not between samples. The FPKM method attempts to account for both feature size and library depth. However, a problem with this approach is that the expression estimate of each gene is dependent on the expression levels of all other genes. As a result, small expression changes between samples in highly expressed genes can skew the estimates for lowly expressed genes, creating artificial differences between samples. Some methods attempt to overcome this by assuming that most genes are not differentially expressed and calculating a per-sample scaling factor. Other methods, attempt to identify a “control set” of consistently expressed features for normalization purposes or make use of “spike-in” reagents of known concentrations. Still others transform read counts

	from each sample to a consistent distribution (e.g., quantile normalization) or use a combination of the above strategies. Many normalization strategies are directly incorporated into the differential expression methods (Cuffdiff, edgeR, DESeq, etc.). No one solution is likely appropriate for all applications or situations. If false positives are a major concern, attempting multiple approaches is desirable. The general topic of RNA-seq data normalization, especially in the context of differential expression analysis, has been extensively reviewed [66-68, 95, 272, 286, 287].
