# Supplementary material for: Informatics for RNA Sequencing: A Web Resource for Analysis on the Cloud
Source: PLoS Comput Biol. 2015 Aug 6;11(8):e1004393. doi: 10.1371/journal.pcbi.1004393 (PMC4527835; doi:10.1371/journal.pcbi.1004393)
Supplement: S8 Table — The following table provides a list of general resources to help understand the background of RNA biology, next-generation sequencing, RNA-seq laboratory methods, and RNA-seq analysis. Additional educational resources can be found in the resources section of the online tutorial at www.rnaseq.wiki. (PDF) [file pcbi.1004393.s010.pdf]

**S8 Table. General resources for RNA-seq analysis**

The following table provides a list of general resources to help understand the background of RNA biology, next generation sequencing, RNA-seq laboratory methods, and RNA-seq analysis. Additional educational resources can be found in the resources section of the online tutorial at www.rnaseq.wiki.

| Resource name and description |
|---|
| SeqAnswers. An online forum for next generation sequencing. |
| BioStars. An online forum for bioinformatics [90]. |
| Illumina videos on the basics of NGS sequencing: video 1, video 2. |
| Molecular Biology of the Cell. From DNA to RNA. A comprehensive introduction to transcription, strandedness, RNA types, gene regulation, RNA polymerase function, splicing, and so on. |
| The RNA-seqlopedia. An overview of RNA-seq and the choices necessary to carry out a successful RNA-seq experiment. |
| RNA-seq Data: Challenges in and Recommendations for Experimental Design and Analysis. |
| RNA Bioinformatics, a 25 chapter book covering many topics relevant to RNA-seq analysis. |
| The RNA-seq blog. An actively maintained blog of RNA-seq related developments. |
| RNA-seq - Protocols and Algorithms. An active RNA-seq blog. |
| HTS Mappers. An actively maintained list of short read aligners (RNA-seq aligners are indicated in red). |
| The periodic table of bioinformatics. A list of commonly used bioinformatics tools. |
| ENCODE standards, guidelines and best practices for RNA-seq. |
| REMC standards and guidelines for RNA-sequencing. |
| A comprehensive assessment of RNA-seq accuracy, reproducibility and information content by the Sequencing Quality Control Consortium. |
| List of RNA-seq bioinformatics tools. |
| GEO: The Gene Expression Omnibus (contains many publicly available RNA-seq data sets). |
