# Supplementary material for: Informatics for RNA Sequencing: A Web Resource for Analysis on the Cloud
Source: PLoS Comput Biol. 2015 Aug 6;11(8):e1004393. doi: 10.1371/journal.pcbi.1004393 (PMC4527835; doi:10.1371/journal.pcbi.1004393)
Supplement: S9 Table — The following table lists RNA-seq workshops and other tutorials complementary to this article. These examples are limited to online materials or short workshops. Not listed here are formal training programs or degrees in bioinformatics. For ongoing discussion of this topic, refer to these BioStar posts: https://www.biostars.org/p/79845/ and https://www.biostars.org/p/11034/. (PDF) [file pcbi.1004393.s011.pdf]

S9 Table. RNA-seq workshops and online tutorials

The following table lists RNA-seq workshops and other tutorials complementary to this article. These examples are limited to online materials or short workshops. Not listed here are formal training programs or degrees in bioinformatics. For ongoing discussion of this topic, refer to these BioStar [90] posts: https://www.biostars.org/p/79845/ and https://www.biostars.org/p/11034/.

**Workshop/Tutorial**
Canadian Bioinformatics Workshops (CBW). Informatics for RNA-sequence analysis. Various additional bioinformatics courses. A live delivery of the tutorials accompanying this publication.
Cold Spring Harbor Laboratory (CSHL). Advanced Sequencing Technologies & Applications.
Michigan State University. Analyzing Next-Generation Sequencing Data.
EBI. Advanced RNA-Seq and ChiP-Seq Data Analysis.
UC Davis Bioinformatics Training Program. RNA-Seq Workshop: From Pipette to P-value! Bootcamp: Introduction to RNA-seq.
EMBL. Various bioinformatics courses.
Wellcome Trust. Various bioinformatics courses.
ECSEQ Bioinformatics. RNA-seq Bioinformatics: A Practical Introduction.
Bioinformatics.org. Various bioinformatics courses.
Data carpentry. Various data science relevant topics.
Software carpentry. Various software and analysis relevant topics.
HarvardX. Case study: RNA-seq data analysis.
Princeton RNA-seq workshop.
