# Supplementary material for: Informatics for RNA Sequencing: A Web Resource for Analysis on the Cloud
Source: PLoS Comput Biol. 2015 Aug 6;11(8):e1004393. doi: 10.1371/journal.pcbi.1004393 (PMC4527835; doi:10.1371/journal.pcbi.1004393)
Supplement: S1 Text — (PDF) [file pcbi.1004393.s012.pdf]

S1 Text. Supplementary References

91. Chandramohan R, Wu PY, Phan JH, Wang MD. Benchmarking RNA-Seq quantification tools. Conference proceedings : Annual International Conference of the IEEE Engineering in Medicine and Biology Society IEEE Engineering in Medicine and Biology Society Annual Conference. 2013;2013:647-50. Epub 2013/10/11. doi: 10.1109/EMBC.2013.6609583. PubMed PMID: 24109770.

92. Steijger T, Abril JF, Engstrom PG, Kokocinski F, Hubbard TJ, Guigo R, et al. Assessment of transcript reconstruction methods for RNA-seq. Nature methods. 2013;10(12):1177-84. Epub 2013/11/05. doi: 10.1038/nmeth.2714. PubMed PMID: 24185837; PubMed Central PMCID: PMC3851240.

93. George NI, Chang CW. DAFS: a data-adaptive flag method for RNA-sequencing data to differentiate genes with low and high expression. BMC bioinformatics. 2014;15:92. Epub 2014/04/02. doi: 10.1186/1471-2105-15-92. PubMed PMID: 24685233; PubMed Central PMCID: PMC4098771.

94. Angelini C, De Canditiis D, De Feis I. Computational approaches for isoform detection and estimation: good and bad news. BMC bioinformatics. 2014;15:135. Epub 2014/06/03. doi: 10.1186/1471-2105-15-135. PubMed PMID: 24885830; PubMed Central PMCID: PMC4098781.

95. Dillies MA, Rau A, Aubert J, Hennequet-Antier C, Jeanmougin M, Servant N, et al. A comprehensive evaluation of normalization methods for Illumina high-throughput RNA sequencing data analysis. Briefings in bioinformatics. 2013;14(6):671-83. Epub 2012/09/19. doi: 10.1093/bib/bbs046. PubMed PMID: 22988256.

96. Pabinger S, Dander A, Fischer M, Snajder R, Sperk M, Efremova M, et al. A survey of tools for variant analysis of next-generation genome sequencing data. Briefings in bioinformatics. 2014;15(2):256-78. Epub 2013/01/24. doi: 10.1093/bib/bbs086. PubMed PMID: 23341494; PubMed Central PMCID: PMC3956068.

97. Castle JC, Loewer M, Boegel S, Tadmor AD, Boisguerin V, de Graaf J, et al. Mutated tumor alleles are expressed according to their DNA frequency. Scientific reports. 2014;4:4743. Epub 2014/04/23. doi: 10.1038/srep04743. PubMed PMID: 24752137; PubMed Central PMCID: PMC3994436.

98. Wang Q, Xia J, Jia P, Pao W, Zhao Z. Application of next generation sequencing to human gene fusion detection: computational tools, features and perspectives. Briefings in bioinformatics. 2013;14(4):506-19. Epub 2012/08/11. doi: 10.1093/bib/bbs044. PubMed PMID: 22877769; PubMed Central PMCID: PMC3713712.

99. Mertens F, Tayebwa J. Evolving techniques for gene fusion detection in soft tissue tumours. Histopathology. 2014;64(1):151-62. Epub 2013/12/11. doi: 10.1111/his.12272. PubMed PMID: 24320890.

100. Kreuze JF, Perez A, Untiveros M, Quispe D, Fuentes S, Barker I, et al. Complete viral genome sequence and discovery of novel viruses by deep sequencing of small RNAs: a generic method for diagnosis, discovery and sequencing of viruses. Virology. 2009;388(1):1-7. Epub 2009/04/28. doi: 10.1016/j.virol.2009.03.024. PubMed PMID: 19394993.

101. Tang KW, Alaei-Mahabadi B, Samuelsson T, Lindh M, Larsson E. The landscape of viral expression and host gene fusion and adaptation in human cancer. Nature communications. 2013;4:2513. Epub 2013/10/03. doi: 10.1038/ncomms3513. PubMed PMID: 24085110; PubMed Central PMCID: PMC3806554.

102. Hooper JE. A survey of software for genome-wide discovery of differential splicing in RNA-Seq data. Human genomics. 2014;8:3. Epub 2014/01/23. doi: 10.1186/1479-7364-8-3. PubMed PMID: 24447644; PubMed Central PMCID: PMC3903050.

103. Li X, Nair A, Wang S, Wang L. Quality Control of RNA-Seq Experiments. Methods in molecular biology. 2015;1269:137-46. Epub 2015/01/13. doi: 10.1007/978-1-4939-2291-8_8. PubMed PMID: 25577376.
104. Yang X, Liu D, Liu F, Wu J, Zou J, Xiao X, et al. HTQC: a fast quality control toolkit for Illumina sequencing data. BMC bioinformatics. 2013;14:33. Epub 2013/02/01. doi: 10.1186/1471-2105-14-33. PubMed PMID: 23363224; PubMed Central PMCID: PMC3571943.
105. Guo Y, Zhao S, Sheng Q, Ye F, Li J, Lehmann B, et al. Multi-perspective quality control of Illumina exome sequencing data using QC3. Genomics. 2014;103(5-6):323-8. Epub 2014/04/08. doi: 10.1016/j.ygeno.2014.03.006. PubMed PMID: 24703969.
106. Anvar SY, Khachatryan L, Vermaat M, van Galen M, Pulyakhina I, Ariyurek Y, et al. Determining the quality and complexity of next-generation sequencing data without a reference genome. Genome biology. 2014;15(12):555. Epub 2014/12/18. doi: 10.1186/s13059-014-0555-3. PubMed PMID: 25514851; PubMed Central PMCID: PMC4298064.
107. Del Fabbro C, Scalabrin S, Morgante M, Giorgi FM. An extensive evaluation of read trimming effects on Illumina NGS data analysis. PloS one. 2013;8(12):e85024. Epub 2014/01/01. doi: 10.1371/journal.pone.0085024. PubMed PMID: 24376861; PubMed Central PMCID: PMC3871669.
108. Dodt M, Roehr JT, Ahmed R, Dieterich C. FLEXBAR-Flexible Barcode and Adapter Processing for Next-Generation Sequencing Platforms. Biology (Basel). 2012;1(3):895-905. doi: 10.3390/biology1030895. PubMed PMID: 24832523; PubMed Central PMCID: PMC4009805.
109. Kim D, Pertea G, Trapnell C, Pimentel H, Kelley R, Salzberg SL. TopHat2: accurate alignment of transcriptomes in the presence of insertions, deletions and gene fusions. Genome biology. 2013;14(4):R36. Epub 2013/04/27. doi: 10.1186/gb-2013-14-4-r36. PubMed PMID: 23618408; PubMed Central PMCID: PMC4053844.
110. Dobin A, Davis CA, Schlesinger F, Drenkow J, Zaleski C, Jha S, et al. STAR: ultrafast universal RNA-seq aligner. Bioinformatics. 2013;29(1):15-21. Epub 2012/10/30. doi: 10.1093/bioinformatics/bts635. PubMed PMID: 23104886; PubMed Central PMCID: PMC3530905.
111. Kim D, Langmead B, Salzberg SL. HISAT: a fast spliced aligner with low memory requirements. Nature methods. 2015;12(4):357-60. doi: 10.1038/nmeth.3317. PubMed PMID: 25751142.
112. Hoffmann S, Otto C, Doose G, Tanzer A, Langenberger D, Christ S, et al. A multi-split mapping algorithm for circular RNA, splicing, trans-splicing and fusion detection. Genome biology. 2014;15(2):R34. doi: 10.1186/gb-2014-15-2-r34. PubMed PMID: 24512684; PubMed Central PMCID: PMC4056463.
113. Wang K, Singh D, Zeng Z, Coleman SJ, Huang Y, Savich GL, et al. MapSplice: accurate mapping of RNA-seq reads for splice junction discovery. Nucleic acids research. 2010;38(18):e178. Epub 2010/08/31. doi: 10.1093/nar/gkq622. PubMed PMID: 20802226; PubMed Central PMCID: PMC2952873.
114. Butterfield YS, Kreitzman M, Thiessen N, Corbett RD, Li Y, Pang J, et al. JAGuaR: junction alignments to genome for RNA-seq reads. PloS one. 2014;9(7):e102398. doi: 10.1371/journal.pone.0102398. PubMed PMID: 25062255; PubMed Central PMCID: PMC4111418.
115. Au KF, Jiang H, Lin L, Xing Y, Wong WH. Detection of splice junctions from paired-end RNA-seq data by SpliceMap. Nucleic acids research. 2010;38(14):4570-8. doi: 10.1093/nar/gkq211. PubMed PMID: 20371516; PubMed Central PMCID: PMC2919714.
116. Dimon MT, Sorber K, DeRisi JL. HMMSplicer: a tool for efficient and sensitive discovery of known and novel splice junctions in RNA-Seq data. PloS one. 2010;5(11):e13875. doi:

10.1371/journal.pone.0013875. PubMed PMID: 21079731; PubMed Central PMCID: PMC2975632.
117. Burns PD, Li Y, Ma J, Borodovsky M. UnSplicer: mapping spliced RNA-Seq reads in compact genomes and filtering noisy splicing. Nucleic acids research. 2014;42(4):e25. doi: 10.1093/nar/gkt1141. PubMed PMID: 24259430; PubMed Central PMCID: PMC3936741.
118. Fonseca NA, Rung J, Brazma A, Marioni JC. Tools for mapping high-throughput sequencing data. Bioinformatics. 2012;28(24):3169-77. Epub 2012/10/13. doi: 10.1093/bioinformatics/bts605. PubMed PMID: 23060614.
119. Hatem A, Bozdag D, Toland AE, Catalyurek UV. Benchmarking short sequence mapping tools. BMC bioinformatics. 2013;14:184. Epub 2013/06/14. doi: 10.1186/1471-2105-14-184. PubMed PMID: 23758764; PubMed Central PMCID: PMC3694458.
120. Langmead B, Trapnell C, Pop M, Salzberg SL. Ultrafast and memory-efficient alignment of short DNA sequences to the human genome. Genome biology. 2009;10(3):R25. doi: 10.1186/gb-2009-10-3-r25. PubMed PMID: 19261174; PubMed Central PMCID: PMC2690996.
121. Li H, Durbin R. Fast and accurate short read alignment with Burrows-Wheeler transform. Bioinformatics. 2009;25(14):1754-60. doi: 10.1093/bioinformatics/btp324. PubMed PMID: 19451168; PubMed Central PMCID: PMC2705234.
122. Kroll KW, Mokaram NE, Pelletier AR, Frankhouser DE, Westphal MS, Stump PA, et al. Quality Control for RNA-Seq (QuaCRS): An Integrated Quality Control Pipeline. Cancer informatics. 2014;13(Suppl 3):7-14. doi: 10.4137/CIN.S14022. PubMed PMID: 25368506; PubMed Central PMCID: PMC4214596.
123. Wang L, Wang S, Li W. RSeQC: quality control of RNA-seq experiments. Bioinformatics. 2012;28(16):2184-5. doi: 10.1093/bioinformatics/bts356. PubMed PMID: 22743226.
124. DeLuca DS, Levin JZ, Sivachenko A, Fennell T, Nazaire MD, Williams C, et al. RNA-SeQC: RNA-seq metrics for quality control and process optimization. Bioinformatics. 2012;28(11):1530-2. doi: 10.1093/bioinformatics/bts196. PubMed PMID: 22539670; PubMed Central PMCID: PMC3356847.
125. Lassmann T, Hayashizaki Y, Daub CO. SAMStat: monitoring biases in next generation sequencing data. Bioinformatics. 2011;27(1):130-1. doi: 10.1093/bioinformatics/btq614. PubMed PMID: 21088025; PubMed Central PMCID: PMC3008642.
126. Cabanski CR, Wilkerson MD, Soloway M, Parker JS, Liu J, Prins JF, et al. BlackOPs: increasing confidence in variant detection through mappability filtering. Nucleic acids research. 2013;41(19):e178. doi: 10.1093/nar/gkt692. PubMed PMID: 23935067; PubMed Central PMCID: PMC3799449.
127. Jones DC, Ruzzo WL, Peng X, Katze MG. A new approach to bias correction in RNA-Seq. Bioinformatics. 2012;28(7):921-8. doi: 10.1093/bioinformatics/bts055. PubMed PMID: 22285831; PubMed Central PMCID: PMC3315719.
128. Schurch NJ, Cole C, Sherstnev A, Song J, Duc C, Storey KG, et al. Improved annotation of 3' untranslated regions and complex loci by combination of strand-specific direct RNA sequencing, RNA-Seq and ESTs. PloS one. 2014;9(4):e94270. Epub 2014/04/12. doi: 10.1371/journal.pone.0094270. PubMed PMID: 24722185; PubMed Central PMCID: PMC3983147.
129. Ortogero N, Hennig GW, Luong D, Yan W. Computer-assisted annotation of small RNA transcriptomes. Methods in molecular biology. 2015;1218:353-64. Epub 2014/10/17. doi: 10.1007/978-1-4939-1538-5_22. PubMed PMID: 25319663.
130. Musacchia F, Basu S, Petrosino G, Salvemini M, Sanges R. Annocript: a flexible pipeline for the annotation of transcriptomes able to identify putative long noncoding RNAs. Bioinformatics. 2015. doi: 10.1093/bioinformatics/btv106. PubMed PMID: 25701574.

131. Zhu Y, Li M, Sousa AM, Sestan N. XSAnno: a framework for building ortholog models in cross-species transcriptome comparisons. BMC genomics. 2014;15:343. doi: 10.1186/1471-2164-15-343. PubMed PMID: 24884593; PubMed Central PMCID: PMC4035071.
132. Lomsadze A, Burns PD, Borodovsky M. Integration of mapped RNA-Seq reads into automatic training of eukaryotic gene finding algorithm. Nucleic acids research. 2014;42(15):e119. doi: 10.1093/nar/gku557. PubMed PMID: 24990371; PubMed Central PMCID: PMC4150757.
133. Sharma P, Mantri SS. WImpiBLAST: web interface for mpiBLAST to help biologists perform large-scale annotation using high performance computing. PloS one. 2014;9(6):e101144. doi: 10.1371/journal.pone.0101144. PubMed PMID: 24979410; PubMed Central PMCID: PMC4076281.
134. Bischler T, Kopf M, Voss B. Transcript mapping based on dRNA-seq data. BMC bioinformatics. 2014;15:122. doi: 10.1186/1471-2105-15-122. PubMed PMID: 24780064; PubMed Central PMCID: PMC4016656.
135. Amman F, Wolfinger MT, Lorenz R, Hofacker IL, Stadler PF, Findeiss S. TSSAR: TSS annotation regime for dRNA-seq data. BMC bioinformatics. 2014;15:89. doi: 10.1186/1471-2105-15-89. PubMed PMID: 24674136; PubMed Central PMCID: PMC4098767.
136. Lu Z, Matera AG. Vicinal: a method for the determination of ncRNA ends using chimeric reads from RNA-seq experiments. Nucleic acids research. 2014;42(9):e79. doi: 10.1093/nar/gku207. PubMed PMID: 24623808; PubMed Central PMCID: PMC4027162.
137. Liu J, Xiao H, Huang S, Li F. OMIGA: Optimized Maker-Based Insect Genome Annotation. Mol Genet Genomics. 2014;289(4):567-73. doi: 10.1007/s00438-014-0831-7. PubMed PMID: 24609470.
138. Ryvkin P, Leung YY, Ungar LH, Gregory BD, Wang LS. Using machine learning and high-throughput RNA sequencing to classify the precursors of small non-coding RNAs. Methods. 2014;67(1):28-35. doi: 10.1016/j.ymeth.2013.10.002. PubMed PMID: 24145223; PubMed Central PMCID: PMC3991776.
139. Jones M, Blaxter M. afterParty: turning raw transcriptomes into permanent resources. BMC bioinformatics. 2013;14:301. doi: 10.1186/1471-2105-14-301. PubMed PMID: 24093729; PubMed Central PMCID: PMC3856601.
140. Axtell MJ. ShortStack: comprehensive annotation and quantification of small RNA genes. Rna. 2013;19(6):740-51. doi: 10.1261/rna.035279.112. PubMed PMID: 23610128; PubMed Central PMCID: PMC3683909.
141. Gao Y, Wang J, Zhao F. CIRI: an efficient and unbiased algorithm for de novo circular RNA identification. Genome biology. 2015;16(1):4. doi: 10.1186/s13059-014-0571-3. PubMed PMID: 25583365; PubMed Central PMCID: PMC4316645.
142. Gomes CP, Cho JH, Hood L, Franco OL, Pereira RW, Wang K. A Review of Computational Tools in microRNA Discovery. Frontiers in genetics. 2013;4:81. Epub 2013/05/31. doi: 10.3389/fgene.2013.00081. PubMed PMID: 23720668; PubMed Central PMCID: PMC3654206.
143. Zhang Z, Jiang L, Wang J, Gu P, Chen M. MTide: an integrated tool for the identification of miRNA-target interaction in plants. Bioinformatics. 2015;31(2):290-1. doi: 10.1093/bioinformatics/btu633. PubMed PMID: 25256573.
144. Hoogstrate Y, Jenster G, Martens-Uzunova ES. FlaiMapper: computational annotation of small ncRNA-derived fragments using RNA-seq high-throughput data. Bioinformatics. 2015;31(5):665-73. doi: 10.1093/bioinformatics/btu696. PubMed PMID: 25338717.
145. An J, Lai J, Sajjanhar A, Lehman ML, Nelson CC. miRPlant: an integrated tool for identification of plant miRNA from RNA sequencing data. BMC bioinformatics. 2014;15:275.

doi: 10.1186/1471-2105-15-275. PubMed PMID: 25117656; PubMed Central PMCID: PMC4141084.
146. Marsico A, Huska MR, Lasserre J, Hu H, Vucicevic D, Musahl A, et al. PROmiRNA: a new miRNA promoter recognition method uncovers the complex regulation of intronic miRNAs. Genome biology. 2013;14(8):R84. doi: 10.1186/gb-2013-14-8-r84. PubMed PMID: 23958307; PubMed Central PMCID: PMC4053815.
147. Muller S, Rycak L, Winter P, Kahl G, Koch I, Rotter B. omiRas: a Web server for differential expression analysis of miRNAs derived from small RNA-Seq data. Bioinformatics. 2013;29(20):2651-2. doi: 10.1093/bioinformatics/btt457. PubMed PMID: 23946503.
148. Alon S, Erew M, Eisenberg E. DREAM: a webserver for the identification of editing sites in mature miRNAs using deep sequencing data. Bioinformatics. 2015. doi: 10.1093/bioinformatics/btv187. PubMed PMID: 25840043.
149. Lu B, Zeng Z, Shi T. Comparative study of de novo assembly and genome-guided assembly strategies for transcriptome reconstruction based on RNA-Seq. Science China Life sciences. 2013;56(2):143-55. Epub 2013/02/09. doi: 10.1007/s11427-013-4442-z. PubMed PMID: 23393030.
150. Guttman M, Garber M, Levin JZ, Donaghey J, Robinson J, Adiconis X, et al. Ab initio reconstruction of cell type-specific transcriptomes in mouse reveals the conserved multi-exonic structure of lincRNAs. Nature biotechnology. 2010;28(5):503-10. doi: 10.1038/nbt.1633. PubMed PMID: 20436462; PubMed Central PMCID: PMC2868100.
151. Pertea M, Pertea GM, Antonescu CM, Chang TC, Mendell JT, Salzberg SL. StringTie enables improved reconstruction of a transcriptome from RNA-seq reads. Nature biotechnology. 2015;33(3):290-5. doi: 10.1038/nbt.3122. PubMed PMID: 25690850.
152. Maretty L, Sibbesen JA, Krogh A. Bayesian transcriptome assembly. Genome biology. 2014;15(10):501. doi: 10.1186/s13059-014-0501-4. PubMed PMID: 25367074; PubMed Central PMCID: PMC4397945.
153. Li W, Feng J, Jiang T. IsoLasso: a LASSO regression approach to RNA-Seq based transcriptome assembly. J Comput Biol. 2011;18(11):1693-707. doi: 10.1089/cmb.2011.0171. PubMed PMID: 21951053; PubMed Central PMCID: PMC3216102.
154. Schliesky S, Gowik U, Weber AP, Brautigam A. RNA-Seq Assembly - Are We There Yet? Frontiers in plant science. 2012;3:220. Epub 2012/10/12. doi: 10.3389/fpls.2012.00220. PubMed PMID: 23056003; PubMed Central PMCID: PMC3457010.
155. Misner I, Bicep C, Lopez P, Halary S, Bapteste E, Lane CE. Sequence comparative analysis using networks: software for evaluating de novo transcript assembly from next-generation sequencing. Molecular biology and evolution. 2013;30(8):1975-86. Epub 2013/05/15. doi: 10.1093/molbev/mst087. PubMed PMID: 23666209; PubMed Central PMCID: PMC3708500.
156. Amin S, Prentis PJ, Gilding EK, Pavasovic A. Assembly and annotation of a non-model gastropod (Nerita melanotragus) transcriptome: a comparison of de novo assemblers. BMC research notes. 2014;7:488. Epub 2014/08/03. doi: 10.1186/1756-0500-7-488. PubMed PMID: 25084827; PubMed Central PMCID: PMC4124492.
157. Finseth FR, Harrison RG. A comparison of next generation sequencing technologies for transcriptome assembly and utility for RNA-Seq in a non-model bird. PloS one. 2014;9(10):e108550. Epub 2014/10/04. doi: 10.1371/journal.pone.0108550. PubMed PMID: 25279728; PubMed Central PMCID: PMC4184788.
158. Janes J, Hu F, Lewin A, Turro E. A comparative study of RNA-seq analysis strategies. Briefings in bioinformatics. 2015. Epub 2015/03/20. doi: 10.1093/bib/bbv007. PubMed PMID: 25788326.

159. Haas BJ, Papanicolaou A, Yassour M, Grabherr M, Blood PD, Bowden J, et al. De novo transcript sequence reconstruction from RNA-seq using the Trinity platform for reference generation and analysis. Nature protocols. 2013;8(8):1494-512. doi: 10.1038/nprot.2013.084. PubMed PMID: 23845962; PubMed Central PMCID: PMC3875132.
160. Schulz MH, Zerbino DR, Vingron M, Birney E. Oases: robust de novo RNA-seq assembly across the dynamic range of expression levels. Bioinformatics. 2012;28(8):1086-92. doi: 10.1093/bioinformatics/bts094. PubMed PMID: 22368243; PubMed Central PMCID: PMC3324515.
161. Li B, Dewey CN. RSEM: accurate transcript quantification from RNA-Seq data with or without a reference genome. BMC bioinformatics. 2011;12:323. doi: 10.1186/1471-2105-12-323. PubMed PMID: 21816040; PubMed Central PMCID: PMC3163565.
162. Le HS, Schulz MH, McCauley BM, Hinman VF, Bar-Joseph Z. Probabilistic error correction for RNA sequencing. Nucleic acids research. 2013;41(10):e109. doi: 10.1093/nar/gkt215. PubMed PMID: 23558750; PubMed Central PMCID: PMC3664804.
163. Bao E, Jiang T, Girke T. BRANCH: boosting RNA-Seq assemblies with partial or related genomic sequences. Bioinformatics. 2013;29(10):1250-9. doi: 10.1093/bioinformatics/btt127. PubMed PMID: 23493323.
164. Chu HT, Hsiao WW, Chen JC, Yeh TJ, Tsai MH, Lin H, et al. EBARDenovo: highly accurate de novo assembly of RNA-Seq with efficient chimera-detection. Bioinformatics. 2013;29(8):1004-10. doi: 10.1093/bioinformatics/btt092. PubMed PMID: 23457040.
165. Chang Z, Li G, Liu J, Zhang Y, Ashby C, Liu D, et al. Bridger: a new framework for de novo transcriptome assembly using RNA-seq data. Genome biology. 2015;16:30. doi: 10.1186/s13059-015-0596-2. PubMed PMID: 25723335; PubMed Central PMCID: PMC4342890.
166. Roberts A, Pachter L. Streaming fragment assignment for real-time analysis of sequencing experiments. Nature methods. 2013;10(1):71-3. doi: 10.1038/nmeth.2251. PubMed PMID: 23160280; PubMed Central PMCID: PMC3880119.
167. Patro R, Mount SM, Kingsford C. Sailfish enables alignment-free isoform quantification from RNA-seq reads using lightweight algorithms. Nature biotechnology. 2014;32(5):462-4. doi: 10.1038/nbt.2862. PubMed PMID: 24752080; PubMed Central PMCID: PMC4077321.
168. Zhang Z, Wang W. RNA-Skim: a rapid method for RNA-Seq quantification at transcript level. Bioinformatics. 2014;30(12):i283-i92. doi: 10.1093/bioinformatics/btu288. PubMed PMID: 24931995; PubMed Central PMCID: PMC4058932.
169. Behr J, Kahles A, Zhong Y, Sreedharan VT, Drewe P, Ratsch G. MITIE: Simultaneous RNA-Seq-based transcript identification and quantification in multiple samples. Bioinformatics. 2013;29(20):2529-38. doi: 10.1093/bioinformatics/btt442. PubMed PMID: 23980025; PubMed Central PMCID: PMC3789545.
170. Mezlini AM, Smith EJ, Fiume M, Buske O, Savich GL, Shah S, et al. iReckon: simultaneous isoform discovery and abundance estimation from RNA-seq data. Genome research. 2013;23(3):519-29. doi: 10.1101/gr.142232.112. PubMed PMID: 23204306; PubMed Central PMCID: PMC3589540.
171. Mangul S, Caciula A, Glebova O, Mandoiu I, Zelikovsky A. Improved transcriptome quantification and reconstruction from RNA-Seq reads using partial annotations. In Silico Biol. 2011;11(5-6):251-61. doi: 10.3233/ISB-2012-0459. PubMed PMID: 23202426.
172. Anders S, Pyl PT, Huber W. HTSeq-a Python framework to work with high-throughput sequencing data. Bioinformatics. 2015;31(2):166-9. Epub 2014/09/28. doi: 10.1093/bioinformatics/btu638. PubMed PMID: 25260700; PubMed Central PMCID: PMC4287950.

173. Liao Y, Smyth GK, Shi W. featureCounts: an efficient general purpose program for assigning sequence reads to genomic features. Bioinformatics. 2014;30(7):923-30. doi: 10.1093/bioinformatics/btt656. PubMed PMID: 24227677.
174. Schmid MW, Grossniklaus U. Rcount: simple and flexible RNA-Seq read counting. Bioinformatics. 2015;31(3):436-7. doi: 10.1093/bioinformatics/btu680. PubMed PMID: 25322836.
175. Finotello F, Lavezzo E, Bianco L, Barzon L, Mazzon P, Fontana P, et al. Reducing bias in RNA sequencing data: a novel approach to compute counts. BMC bioinformatics. 2014;15 Suppl 1:S7. doi: 10.1186/1471-2105-15-S1-S7. PubMed PMID: 24564404; PubMed Central PMCID: PMC4016203.
176. Hashimoto TB, Edwards MD, Gifford DK. Universal count correction for high-throughput sequencing. PLoS computational biology. 2014;10(3):e1003494. doi: 10.1371/journal.pcbi.1003494. PubMed PMID: 24603409; PubMed Central PMCID: PMC3945112.
177. Sharma CM, Vogel J. Differential RNA-seq: the approach behind and the biological insight gained. Current opinion in microbiology. 2014;19:97-105. Epub 2014/07/16. doi: 10.1016/j.mib.2014.06.010. PubMed PMID: 25024085.
178. Ritchie ME, Phipson B, Wu D, Hu Y, Law CW, Shi W, et al. limma powers differential expression analyses for RNA-sequencing and microarray studies. Nucleic acids research. 2015;43(7):e47. doi: 10.1093/nar/gkv007. PubMed PMID: 25605792.
179. Love MI, Huber W, Anders S. Moderated estimation of fold change and dispersion for RNA-seq data with DESeq2. Genome biology. 2014;15(12):550. doi: 10.1186/s13059-014-0550-8. PubMed PMID: 25516281; PubMed Central PMCID: PMC4302049.
180. Robinson MD, McCarthy DJ, Smyth GK. edgeR: a Bioconductor package for differential expression analysis of digital gene expression data. Bioinformatics. 2010;26(1):139-40. doi: 10.1093/bioinformatics/btp616. PubMed PMID: 19910308; PubMed Central PMCID: PMC2796818.
181. Davidson NM, Oshlack A. Corset: enabling differential gene expression analysis for de novo assembled transcriptomes. Genome biology. 2014;15(7):410. doi: 10.1186/s13059-014-0410-6. PubMed PMID: 25063469; PubMed Central PMCID: PMC4165373.
182. Yu D, Huber W, Vitek O. Shrinkage estimation of dispersion in Negative Binomial models for RNA-seq experiments with small sample size. Bioinformatics. 2013;29(10):1275-82. doi: 10.1093/bioinformatics/btt143. PubMed PMID: 23589650; PubMed Central PMCID: PMC3654711.
183. Gu J, Wang X, Halakivi-Clarke L, Clarke R, Xuan J. BADGE: a novel Bayesian model for accurate abundance quantification and differential analysis of RNA-Seq data. BMC bioinformatics. 2014;15 Suppl 9:S6. doi: 10.1186/1471-2105-15-S9-S6. PubMed PMID: 25252852; PubMed Central PMCID: PMC4168709.
184. Soneson C. compcodeR--an R package for benchmarking differential expression methods for RNA-seq data. Bioinformatics. 2014;30(17):2517-8. doi: 10.1093/bioinformatics/btu324. PubMed PMID: 24813215.
185. Rau A, Marot G, Jaffrezic F. Differential meta-analysis of RNA-seq data from multiple studies. BMC bioinformatics. 2014;15:91. doi: 10.1186/1471-2105-15-91. PubMed PMID: 24678608; PubMed Central PMCID: PMC4021464.
186. Clark NR, Hu KS, Feldmann AS, Kou Y, Chen EY, Duan Q, et al. The characteristic direction: a geometrical approach to identify differentially expressed genes. BMC bioinformatics. 2014;15:79. doi: 10.1186/1471-2105-15-79. PubMed PMID: 24650281; PubMed Central PMCID: PMC4000056.

187. Bi Y, Davuluri RV. NPEBseq: nonparametric empirical bayesian-based procedure for differential expression analysis of RNA-seq data. BMC bioinformatics. 2013;14:262. doi: 10.1186/1471-2105-14-262. PubMed PMID: 23981227; PubMed Central PMCID: PMC3765716.
188. Chen L. Statistical and Computational Methods for High-Throughput Sequencing Data Analysis of Alternative Splicing. Statistics in biosciences. 2013;5(1):138-55. Epub 2013/09/24. doi: 10.1007/s12561-012-9064-7. PubMed PMID: 24058384; PubMed Central PMCID: PMC3776476.
189. Alamancos GP, Agirre E, Eyras E. Methods to study splicing from high-throughput RNA sequencing data. Methods in molecular biology. 2014;1126:357-97. Epub 2014/02/20. doi: 10.1007/978-1-62703-980-2_26. PubMed PMID: 24549677.
190. Li HD, Menon R, Omenn GS, Guan Y. The emerging era of genomic data integration for analyzing splice isoform function. Trends in genetics : TIG. 2014;30(8):340-7. Epub 2014/06/22. doi: 10.1016/j.tig.2014.05.005. PubMed PMID: 24951248; PubMed Central PMCID: PMC4112133.
191. Liu R, Loraine AE, Dickerson JA. Comparisons of computational methods for differential alternative splicing detection using RNA-seq in plant systems. BMC bioinformatics. 2014;15(1):364. Epub 2014/12/17. doi: 10.1186/s12859-014-0364-4. PubMed PMID: 25511303; PubMed Central PMCID: PMC4271460.
192. Anders S, Reyes A, Huber W. Detecting differential usage of exons from RNA-seq data. Genome research. 2012;22(10):2008-17. doi: 10.1101/gr.133744.111. PubMed PMID: 22722343; PubMed Central PMCID: PMC3460195.
193. Niu L, Huang W, Umbach DM, Li L. IUTA: a tool for effectively detecting differential isoform usage from RNA-Seq data. BMC genomics. 2014;15:862. doi: 10.1186/1471-2164-15-862. PubMed PMID: 25283306; PubMed Central PMCID: PMC4195885.
194. Gatto A, Torroja-Fungairino C, Mazzarotto F, Cook SA, Barton PJ, Sanchez-Cabo F, et al. FineSplice, enhanced splice junction detection and quantification: a novel pipeline based on the assessment of diverse RNA-Seq alignment solutions. Nucleic acids research. 2014;42(8):e71. doi: 10.1093/nar/gku166. PubMed PMID: 24574529; PubMed Central PMCID: PMC4005686.
195. Hu Y, Liu Y, Mao X, Jia C, Ferguson JF, Xue C, et al. PennSeq: accurate isoform-specific gene expression quantification in RNA-Seq by modeling non-uniform read distribution. Nucleic acids research. 2014;42(3):e20. doi: 10.1093/nar/gkt1304. PubMed PMID: 24362841; PubMed Central PMCID: PMC3919567.
196. Bernard E, Jacob L, Mairal J, Vert JP. Efficient RNA isoform identification and quantification from RNA-Seq data with network flows. Bioinformatics. 2014;30(17):2447-55. doi: 10.1093/bioinformatics/btu317. PubMed PMID: 24813214; PubMed Central PMCID: PMC4147886.
197. Mudvari P, Movassagh M, Kowsari K, Seyfi A, Kokkinaki M, Edwards NJ, et al. SNPlice: variants that modulate Intron retention from RNA-sequencing data. Bioinformatics. 2015;31(8):1191-8. doi: 10.1093/bioinformatics/btu804. PubMed PMID: 25481010; PubMed Central PMCID: PMC4393518.
198. Vitting-Seerup K, Porse BT, Sandelin A, Waage J. spliceR: an R package for classification of alternative splicing and prediction of coding potential from RNA-seq data. BMC bioinformatics. 2014;15:81. doi: 10.1186/1471-2105-15-81. PubMed PMID: 24655717; PubMed Central PMCID: PMC3998036.
199. Park JW, Tokheim C, Shen S, Xing Y. Identifying differential alternative splicing events from RNA sequencing data using RNASeq-MATS. Methods in molecular biology. 2013;1038:171-9. doi: 10.1007/978-1-62703-514-9_10. PubMed PMID: 23872975.

200. Aschoff M, Hotz-Wagenblatt A, Glatting KH, Fischer M, Eils R, Konig R. SplicingCompass: differential splicing detection using RNA-seq data. Bioinformatics. 2013;29(9):1141-8. doi: 10.1093/bioinformatics/btt101. PubMed PMID: 23449093.
201. Hu Y, Huang Y, Du Y, Orellana CF, Singh D, Johnson AR, et al. DiffSplice: the genome-wide detection of differential splicing events with RNA-seq. Nucleic acids research. 2013;41(2):e39. doi: 10.1093/nar/gks1026. PubMed PMID: 23155066; PubMed Central PMCID: PMC3553996.
202. Kimes PK, Cabanski CR, Wilkerson MD, Zhao N, Johnson AR, Perou CM, et al. SigFuge: single gene clustering of RNA-seq reveals differential isoform usage among cancer samples. Nucleic acids research. 2014;42(14):e113. doi: 10.1093/nar/gku521. PubMed PMID: 25030904; PubMed Central PMCID: PMC4132703.
203. Pulyakhina I, Gazzoli I, t Hoen PA, Verwey N, Dunnen JD, Aartsma-Rus A, et al. SplicePie: a novel analytical approach for the detection of alternative, non-sequential and recursive splicing. Nucleic acids research. 2015. doi: 10.1093/nar/gkv242. PubMed PMID: 25800735.
204. Jia C, Hu Y, Liu Y, Li M. Mapping Splicing Quantitative Trait Loci in RNA-Seq. Cancer informatics. 2015;14(Suppl 1):45-53. Epub 2015/03/04. doi: 10.4137/CIN.S24832. PubMed PMID: 25733796; PubMed Central PMCID: PMC4333812.
205. McKenna A, Hanna M, Banks E, Sivachenko A, Cibulskis K, Kernytsky A, et al. The Genome Analysis Toolkit: a MapReduce framework for analyzing next-generation DNA sequencing data. Genome research. 2010;20(9):1297-303. doi: 10.1101/gr.107524.110. PubMed PMID: 20644199; PubMed Central PMCID: PMC2928508.
206. Goya R, Sun MG, Morin RD, Leung G, Ha G, Wiegand KC, et al. SNVMix: predicting single nucleotide variants from next-generation sequencing of tumors. Bioinformatics. 2010;26(6):730-6. doi: 10.1093/bioinformatics/btq040. PubMed PMID: 20130035; PubMed Central PMCID: PMC2832826.
207. Tang X, Baheti S, Shameer K, Thompson KJ, Wills Q, Niu N, et al. The eSNV-detect: a computational system to identify expressed single nucleotide variants from transcriptome sequencing data. Nucleic acids research. 2014;42(22):e172. doi: 10.1093/nar/gku1005. PubMed PMID: 25352556; PubMed Central PMCID: PMC4267611.
208. Wang C, Davila JI, Baheti S, Bhagwate AV, Wang X, Kocher JP, et al. RVboost: RNA-seq variants prioritization using a boosting method. Bioinformatics. 2014;30(23):3414-6. doi: 10.1093/bioinformatics/btu577. PubMed PMID: 25170027; PubMed Central PMCID: PMC4296157.
209. Monlong J, Calvo M, Ferreira PG, Guigo R. Identification of genetic variants associated with alternative splicing using sQTLseekeR. Nature communications. 2014;5:4698. doi: 10.1038/ncomms5698. PubMed PMID: 25140736; PubMed Central PMCID: PMC4143934.
210. Peralta M, Combes MC, Cenci A, Lashermes P, Dereeper A. SNiPloid: A Utility to Exploit High-Throughput SNP Data Derived from RNA-Seq in Allopolyploid Species. Int J Plant Genomics. 2013;2013:890123. doi: 10.1155/2013/890123. PubMed PMID: 24163691; PubMed Central PMCID: PMC3791807.
211. Nijveen H, van Kaauwen M, Esselink DG, Hoegen B, Vosman B. QualitySNPng: a user-friendly SNP detection and visualization tool. Nucleic acids research. 2013;41(Web Server issue):W587-90. doi: 10.1093/nar/gkt333. PubMed PMID: 23632165; PubMed Central PMCID: PMC3692117.
212. Miller AC, Obholzer ND, Shah AN, Megason SG, Moens CB. RNA-seq-based mapping and candidate identification of mutations from forward genetic screens. Genome research. 2013;23(4):679-86. doi: 10.1101/gr.147322.112. PubMed PMID: 23299976; PubMed Central PMCID: PMC3613584.

213. Philippe N, Salson M, Commes T, Rivals E. CRAC: an integrated approach to the analysis of RNA-seq reads. Genome biology. 2013;14(3):R30. doi: 10.1186/gb-2013-14-3-r30. PubMed PMID: 23537109; PubMed Central PMCID: PMC4053775.
214. Radenbaugh AJ, Ma S, Ewing A, Stuart JM, Collisson EA, Zhu J, et al. RADIA: RNA and DNA integrated analysis for somatic mutation detection. PloS one. 2014;9(11):e111516. doi: 10.1371/journal.pone.0111516. PubMed PMID: 25405470; PubMed Central PMCID: PMC4236012.
215. Ramaswami G, Zhang R, Piskol R, Keegan LP, Deng P, O'Connell MA, et al. Identifying RNA editing sites using RNA sequencing data alone. Nature methods. 2013;10(2):128-32. Epub 2013/01/08. doi: 10.1038/nmeth.2330. PubMed PMID: 23291724; PubMed Central PMCID: PMC3676881.
216. Lee JH, Ang JK, Xiao X. Analysis and design of RNA sequencing experiments for identifying RNA editing and other single-nucleotide variants. Rna. 2013;19(6):725-32. Epub 2013/04/20. doi: 10.1261/rna.037903.112. PubMed PMID: 23598527; PubMed Central PMCID: PMC3683905.
217. Nigita G, Veneziano D, Ferro A. A-to-I RNA Editing: Current Knowledge Sources and Computational Approaches with Special Emphasis on Non-Coding RNA Molecules. Frontiers in bioengineering and biotechnology. 2015;3:37. Epub 2015/04/11. doi: 10.3389/fbioe.2015.00037. PubMed PMID: 25859542; PubMed Central PMCID: PMC4373398.
218. Picardi E, Pesole G. REDItools: high-throughput RNA editing detection made easy. Bioinformatics. 2013;29(14):1813-4. doi: 10.1093/bioinformatics/btt287. PubMed PMID: 23742983.
219. Zhang Q, Xiao X. Genome sequence-independent identification of RNA editing sites. Nature methods. 2015;12(4):347-50. doi: 10.1038/nmeth.3314. PubMed PMID: 25730491; PubMed Central PMCID: PMC4382388.
220. Suzuki T, Ueda H, Okada S, Sakurai M. Transcriptome-wide identification of adenosine-to-inosine editing using the ICE-seq method. Nature protocols. 2015;10(5):715-32. doi: 10.1038/nprot.2015.037. PubMed PMID: 25855956.
221. Stevenson KR, Coolon JD, Wittkopp PJ. Sources of bias in measures of allele-specific expression derived from RNA-sequence data aligned to a single reference genome. BMC genomics. 2013;14:536. Epub 2013/08/08. doi: 10.1186/1471-2164-14-536. PubMed PMID: 23919664; PubMed Central PMCID: PMC3751238.
222. Liu Z, Yang J, Xu H, Li C, Wang Z, Li Y, et al. Comparing computational methods for identification of allele-specific expression based on next generation sequencing data. Genetic epidemiology. 2014;38(7):591-8. Epub 2014/09/04. doi: 10.1002/gepi.21846. PubMed PMID: 25183311.
223. Leon-Novelo LG, McIntyre LM, Fear JM, Graze RM. A flexible Bayesian method for detecting allelic imbalance in RNA-seq data. BMC genomics. 2014;15:920. Epub 2014/10/24. doi: 10.1186/1471-2164-15-920. PubMed PMID: 25339465; PubMed Central PMCID: PMC4230747.
224. Pandey RV, Franssen SU, Futschik A, Schlotterer C. Allelic imbalance metre (Allim), a new tool for measuring allele-specific gene expression with RNA-seq data. Mol Ecol Resour. 2013;13(4):740-5. doi: 10.1111/1755-0998.12110. PubMed PMID: 23615333; PubMed Central PMCID: PMC3739924.
225. Pirinen M, Lappalainen T, Zaitlen NA, Consortium GT, Dermitzakis ET, Donnelly P, et al. Assessing allele-specific expression across multiple tissues from RNA-seq read data. Bioinformatics. 2015. doi: 10.1093/bioinformatics/btv074. PubMed PMID: 25819081.
226. Mayba O, Gilbert HN, Liu J, Haverty PM, Jhunjhunwala S, Jiang Z, et al. MBASED: allele-specific expression detection in cancer tissues and cell lines. Genome biology.

2014;15(8):405. doi: 10.1186/s13059-014-0405-3. PubMed PMID: 25315065; PubMed Central PMCID: PMC4165366.
227. Chen Y, Yao H, Thompson EJ, Tannir NM, Weinstein JN, Su X. VirusSeq: software to identify viruses and their integration sites using next-generation sequencing of human cancer tissue. Bioinformatics. 2013;29(2):266-7. doi: 10.1093/bioinformatics/bts665. PubMed PMID: 23162058; PubMed Central PMCID: PMC3546792.
228. Wang Q, Jia P, Zhao Z. VirusFinder: software for efficient and accurate detection of viruses and their integration sites in host genomes through next generation sequencing data. PloS one. 2013;8(5):e64465. doi: 10.1371/journal.pone.0064465. PubMed PMID: 23717618; PubMed Central PMCID: PMC3663743.
229. Xu G, Strong MJ, Lacey MR, Baribault C, Flemington EK, Taylor CM. RNA CoMPASS: a dual approach for pathogen and host transcriptome analysis of RNA-seq datasets. PloS one. 2014;9(2):e89445. doi: 10.1371/journal.pone.0089445. PubMed PMID: 24586784; PubMed Central PMCID: PMC3934900.
230. Fernandez-Cuesta L, Sun R, Menon R, George J, Lorenz S, Meza-Zepeda LA, et al. Identification of novel fusion genes in lung cancer using breakpoint assembly of transcriptome sequencing data. Genome biology. 2015;16:7. doi: 10.1186/s13059-014-0558-0. PubMed PMID: 25650807; PubMed Central PMCID: PMC4300615.
231. Yorukoglu D, Hach F, Swanson L, Collins CC, Birol I, Sahinalp SC. Dissect: detection and characterization of novel structural alterations in transcribed sequences. Bioinformatics. 2012;28(12):i179-87. doi: 10.1093/bioinformatics/bts214. PubMed PMID: 22689759; PubMed Central PMCID: PMC3371846.
232. Torres-Garcia W, Zheng S, Sivachenko A, Vegesna R, Wang Q, Yao R, et al. PRADA: pipeline for RNA sequencing data analysis. Bioinformatics. 2014;30(15):2224-6. doi: 10.1093/bioinformatics/btu169. PubMed PMID: 24695405; PubMed Central PMCID: PMC4103589.
233. Abate F, Zairis S, Ficarra E, Acquaviva A, Wiggins CH, Frattini V, et al. Pegasus: a comprehensive annotation and prediction tool for detection of driver gene fusions in cancer. BMC Syst Biol. 2014;8(1):97. doi: 10.1186/s12918-014-0097-z. PubMed PMID: 25183062; PubMed Central PMCID: PMC4363948.
234. Iyer MK, Chinnaiyan AM, Maher CA. ChimeraScan: a tool for identifying chimeric transcription in sequencing data. Bioinformatics. 2011;27(20):2903-4. doi: 10.1093/bioinformatics/btr467. PubMed PMID: 21840877; PubMed Central PMCID: PMC3187648.
235. Kim D, Salzberg SL. TopHat-Fusion: an algorithm for discovery of novel fusion transcripts. Genome biology. 2011;12(8):R72. doi: 10.1186/gb-2011-12-8-r72. PubMed PMID: 21835007; PubMed Central PMCID: PMC3245612.
236. Chen K, Wallis JW, Kandoth C, Kalicki-Veizer JM, Mungall KL, Mungall AJ, et al. BreakFusion: targeted assembly-based identification of gene fusions in whole transcriptome paired-end sequencing data. Bioinformatics. 2012;28(14):1923-4. doi: 10.1093/bioinformatics/bts272. PubMed PMID: 22563071; PubMed Central PMCID: PMC3389765.
237. McPherson A, Hormozdiari F, Zayed A, Giuliany R, Ha G, Sun MG, et al. deFuse: an algorithm for gene fusion discovery in tumor RNA-Seq data. PLoS computational biology. 2011;7(5):e1001138. doi: 10.1371/journal.pcbi.1001138. PubMed PMID: 21625565; PubMed Central PMCID: PMC3098195.
238. Li Y, Chien J, Smith DI, Ma J. FusionHunter: identifying fusion transcripts in cancer using paired-end RNA-seq. Bioinformatics. 2011;27(12):1708-10. doi: 10.1093/bioinformatics/btr265. PubMed PMID: 21546395.

239. Benelli M, Pescucci C, Marseglia G, Severgnini M, Torricelli F, Magi A. Discovering chimeric transcripts in paired-end RNA-seq data by using EricScript. Bioinformatics. 2012;28(24):3232-9. doi: 10.1093/bioinformatics/bts617. PubMed PMID: 23093608.
240. Swanson L, Robertson G, Mungall KL, Butterfield YS, Chiu R, Corbett RD, et al. Barnacle: detecting and characterizing tandem duplications and fusions in transcriptome assemblies. BMC genomics. 2013;14:550. doi: 10.1186/1471-2164-14-550. PubMed PMID: 23941359; PubMed Central PMCID: PMC3751903.
241. Abate F, Acquaviva A, Paciello G, Foti C, Ficarra E, Ferrarini A, et al. Bellerophontes: an RNA-Seq data analysis framework for chimeric transcripts discovery based on accurate fusion model. Bioinformatics. 2012;28(16):2114-21. doi: 10.1093/bioinformatics/bts334. PubMed PMID: 22711792.
242. Kalyana-Sundaram S, Shanmugam A, Chinnaiyan AM. Gene Fusion Markup Language: a prototype for exchanging gene fusion data. BMC bioinformatics. 2012;13:269. doi: 10.1186/1471-2105-13-269. PubMed PMID: 23072312; PubMed Central PMCID: PMC3607969.
243. Gulledge AA, Vora H, Patel K, Loraine AE. A protocol for visual analysis of alternative splicing in RNA-Seq data using integrated genome browser. Methods in molecular biology. 2014;1158:123-37. Epub 2014/05/06. doi: 10.1007/978-1-4939-0700-7_8. PubMed PMID: 24792048; PubMed Central PMCID: PMC4070736.
244. Loraine AE, Blakley IC, Jagadeesan S, Harper J, Miller G, Firon N. Analysis and Visualization of RNA-Seq Expression Data Using RStudio, Bioconductor, and Integrated Genome Browser. Methods in molecular biology. 2015;1284:481-501. Epub 2015/03/12. doi: 10.1007/978-1-4939-2444-8_24. PubMed PMID: 25757788; PubMed Central PMCID: PMC4387895.
245. Liu Q, Chen C, Shen E, Zhao F, Sun Z, Wu J. Detection, annotation and visualization of alternative splicing from RNA-Seq data with SplicingViewer. Genomics. 2012;99(3):178-82. doi: 10.1016/j.ygeno.2011.12.003. PubMed PMID: 22226708.
246. Tokheim C, Park JW, Xing Y. PrimerSeq: Design and visualization of RT-PCR primers for alternative splicing using RNA-seq data. Genomics, proteomics & bioinformatics. 2014;12(2):105-9. doi: 10.1016/j.gpb.2014.04.001. PubMed PMID: 24747190.
247. Foissac S, Sammeth M. Analysis of alternative splicing events in custom gene datasets by AStalavista. Methods in molecular biology. 2015;1269:379-92. doi: 10.1007/978-1-4939-2291-8_24. PubMed PMID: 25577392.
248. Krzywinski M, Schein J, Birol I, Connors J, Gascoyne R, Horsman D, et al. Circos: an information aesthetic for comparative genomics. Genome research. 2009;19(9):1639-45. doi: 10.1101/gr.092759.109. PubMed PMID: 19541911; PubMed Central PMCID: PMC2752132.
249. Chelaru F, Smith L, Goldstein N, Bravo HC. Epiviz: interactive visual analytics for functional genomics data. Nature methods. 2014;11(9):938-40. doi: 10.1038/nmeth.3038. PubMed PMID: 25086505; PubMed Central PMCID: PMC4149593.
250. Mariette J, Noirot C, Nabihoudine I, Bardou P, Hoede C, Djari A, et al. RNAbrowse: RNA-Seq de novo assembly results browser. PloS one. 2014;9(5):e96821. doi: 10.1371/journal.pone.0096821. PubMed PMID: 24823498; PubMed Central PMCID: PMC4019526.
251. Severin J, Lizio M, Harshbarger J, Kawaji H, Daub CO, Hayashizaki Y, et al. Interactive visualization and analysis of large-scale sequencing datasets using ZENBU. Nature biotechnology. 2014;32(3):217-9. doi: 10.1038/nbt.2840. PubMed PMID: 24727769.
252. Roge X, Zhang X. RNAseqViewer: visualization tool for RNA-Seq data. Bioinformatics. 2014;30(6):891-2. doi: 10.1093/bioinformatics/btt649. PubMed PMID: 24215023.

253. Watson M, Schnettler E, Kohl A. viRome: an R package for the visualization and analysis of viral small RNA sequence datasets. Bioinformatics. 2013;29(15):1902-3. doi: 10.1093/bioinformatics/btt297. PubMed PMID: 23709497; PubMed Central PMCID: PMC3712215.
254. Jang I, Chang H, Jun Y, Park S, Yang JO, Lee B, et al. miRseqViewer: multi-panel visualization of sequence, structure and expression for analysis of microRNA sequencing data. Bioinformatics. 2015;31(4):596-8. doi: 10.1093/bioinformatics/btu676. PubMed PMID: 25322835.
255. Crabtree J, Agrawal S, Mahurkar A, Myers GS, Rasko DA, White O. Circleator: flexible circular visualization of genome-associated data with BioPerl and SVG. Bioinformatics. 2014;30(21):3125-7. doi: 10.1093/bioinformatics/btu505. PubMed PMID: 25075113; PubMed Central PMCID: PMC4201160.
256. An J, Lai J, Wood DL, Sajjanhar A, Wang C, Tevz G, et al. RNASeqBrowser: A genome browser for simultaneous visualization of raw strand specific RNAseq reads and UCSC genome browser custom tracks. BMC genomics. 2015;16:145. doi: 10.1186/s12864-015-1346-2. PubMed PMID: 25766521; PubMed Central PMCID: PMC4355470.
257. Shirley BC, Mucaki EJ, Whitehead T, Costea PI, Akan P, Rogan PK. Interpretation, stratification and evidence for sequence variants affecting mRNA splicing in complete human genome sequences. Genomics, proteomics & bioinformatics. 2013;11(2):77-85. Epub 2013/03/19. doi: 10.1016/j.gpb.2013.01.008. PubMed PMID: 23499923.
258. Viner C, Dorman SN, Shirley BC, Rogan PK. Validation of predicted mRNA splicing mutations using high-throughput transcriptome data. F1000Res. 2014;3:8. doi: 10.12688/f1000research.3-8.v2. PubMed PMID: 24741438; PubMed Central PMCID: PMC3983938.
259. Spinelli R, Pirola A, Redaelli S, Sharma N, Raman H, Valletta S, et al. Identification of novel point mutations in splicing sites integrating whole-exome and RNA-seq data in myeloproliferative diseases. Mol Genet Genomic Med. 2013;1(4):246-59. doi: 10.1002/mgg3.23. PubMed PMID: 24498620; PubMed Central PMCID: PMC3865592.
260. McPherson A, Wu C, Wyatt AW, Shah S, Collins C, Sahinalp SC. nFuse: discovery of complex genomic rearrangements in cancer using high-throughput sequencing. Genome research. 2012;22(11):2250-61. doi: 10.1101/gr.136572.111. PubMed PMID: 22745232; PubMed Central PMCID: PMC3483554.
261. Chen EA, Souaiaia T, Herstein JS, Evgrafov OV, Spitsyna VN, Rebolini DF, et al. Effect of RNA integrity on uniquely mapped reads in RNA-Seq. BMC research notes. 2014;7:753. Epub 2014/10/24. doi: 10.1186/1756-0500-7-753. PubMed PMID: 25339126; PubMed Central PMCID: PMC4213542.
262. Gallego Romero I, Pai AA, Tung J, Gilad Y. RNA-seq: impact of RNA degradation on transcript quantification. BMC biology. 2014;12:42. Epub 2014/06/03. doi: 10.1186/1741-7007-12-42. PubMed PMID: 24885439; PubMed Central PMCID: PMC4071332.
263. Kumar R, Ichihashi Y, Kimura S, Chitwood DH, Headland LR, Peng J, et al. A High-Throughput Method for Illumina RNA-Seq Library Preparation. Frontiers in plant science. 2012;3:202. Epub 2012/09/14. doi: 10.3389/fpls.2012.00202. PubMed PMID: 22973283; PubMed Central PMCID: PMC3428589.
264. Bhargava V, Head SR, Ordoukhanian P, Mercola M, Subramaniam S. Technical variations in low-input RNA-seq methodologies. Scientific reports. 2014;4:3678. Epub 2014/01/15. doi: 10.1038/srep03678. PubMed PMID: 24419370; PubMed Central PMCID: PMC3890974.
265. Christodoulou DC, Gorham JM, Herman DS, Seidman JG. Construction of normalized RNA-seq libraries for next-generation sequencing using the crab duplex-specific nuclease.

Current protocols in molecular biology / edited by Frederick M Ausubel [et al]. 2011;Chapter 4:Unit4 12. Epub 2011/04/08. doi: 10.1002/0471142727.mb0412s94. PubMed PMID: 21472699; PubMed Central PMCID: PMC3152986.
266. Cabanski CR, Magrini V, Griffith M, Griffith OL, McGrath S, Zhang J, et al. cDNA hybrid capture improves transcriptome analysis on low-input and archived samples. The Journal of molecular diagnostics : JMD. 2014;16(4):440-51. Epub 2014/05/13. doi: 10.1016/j.jmoldx.2014.03.004. PubMed PMID: 24814956; PubMed Central PMCID: PMC4078367.
267. Norton N, Sun Z, Asmann YW, Serie DJ, Necela BM, Bhagwate A, et al. Gene expression, single nucleotide variant and fusion transcript discovery in archival material from breast tumors. PloS one. 2013;8(11):e81925. Epub 2013/11/28. doi: 10.1371/journal.pone.0081925. PubMed PMID: 24278466; PubMed Central PMCID: PMC3838386.
268. Ma Y, Ambannavar R, Stephans J, Jeong J, Dei Rossi A, Liu ML, et al. Fusion transcript discovery in formalin-fixed paraffin-embedded human breast cancer tissues reveals a link to tumor progression. PloS one. 2014;9(4):e94202. Epub 2014/04/15. doi: 10.1371/journal.pone.0094202. PubMed PMID: 24727804; PubMed Central PMCID: PMC3984112.
269. Busby MA, Stewart C, Miller CA, Grzeda KR, Marth GT. Scotty: a web tool for designing RNA-Seq experiments to measure differential gene expression. Bioinformatics. 2013;29(5):656-7. Epub 2013/01/15. doi: 10.1093/bioinformatics/btt015. PubMed PMID: 23314327; PubMed Central PMCID: PMC3582267.
270. Fu GK, Xu W, Wilhelmy J, Mindrinos MN, Davis RW, Xiao W, et al. Molecular indexing enables quantitative targeted RNA sequencing and reveals poor efficiencies in standard library preparations. Proceedings of the National Academy of Sciences of the United States of America. 2014;111(5):1891-6. Epub 2014/01/23. doi: 10.1073/pnas.1323732111. PubMed PMID: 24449890; PubMed Central PMCID: PMC3918775.
271. Jiang L, Schlesinger F, Davis CA, Zhang Y, Li R, Salit M, et al. Synthetic spike-in standards for RNA-seq experiments. Genome research. 2011;21(9):1543-51. Epub 2011/08/06. doi: 10.1101/gr.121095.111. PubMed PMID: 21816910; PubMed Central PMCID: PMC3166838.
272. Katayama S, Tohonen V, Linnarsson S, Kere J. SAMstrt: statistical test for differential expression in single-cell transcriptome with spike-in normalization. Bioinformatics. 2013;29(22):2943-5. Epub 2013/09/03. doi: 10.1093/bioinformatics/btt511. PubMed PMID: 23995393; PubMed Central PMCID: PMC3810855.
273. Mardis ER. Next-generation DNA sequencing methods. Annual review of genomics and human genetics. 2008;9:387-402. Epub 2008/06/26. doi: 10.1146/annurev.genom.9.081307.164359. PubMed PMID: 18576944.
274. Mardis ER. Next-generation sequencing platforms. Annual review of analytical chemistry. 2013;6:287-303. Epub 2013/04/09. doi: 10.1146/annurev-anchem-062012-092628. PubMed PMID: 23560931.
275. Grada A, Weinbrecht K. Next-generation sequencing: methodology and application. The Journal of investigative dermatology. 2013;133(8):e11. Epub 2013/07/17. doi: 10.1038/jid.2013.248. PubMed PMID: 23856935.
276. van Dijk EL, Auger H, Jaszczyszyn Y, Thermes C. Ten years of next-generation sequencing technology. Trends in genetics : TIG. 2014;30(9):418-26. Epub 2014/08/12. doi: 10.1016/j.tig.2014.07.001. PubMed PMID: 25108476.
277. Liu Y, Zhou J, White KP. RNA-seq differential expression studies: more sequence or more replication? Bioinformatics. 2014;30(3):301-4. Epub 2013/12/10. doi:

10.1093/bioinformatics/btt688. PubMed PMID: 24319002; PubMed Central PMCID: PMC3904521.
278. Sims D, Sudbery I, Ilott NE, Heger A, Ponting CP. Sequencing depth and coverage: key considerations in genomic analyses. Nature reviews Genetics. 2014;15(2):121-32. Epub 2014/01/18. doi: 10.1038/nrg3642. PubMed PMID: 24434847.
279. Roberts A, Trapnell C, Donaghey J, Rinn JL, Pachter L. Improving RNA-Seq expression estimates by correcting for fragment bias. Genome biology. 2011;12(3):R22. Epub 2011/03/18. doi: 10.1186/gb-2011-12-3-r22. PubMed PMID: 21410973; PubMed Central PMCID: PMC3129672.
280. Wu TD, Nacu S. Fast and SNP-tolerant detection of complex variants and splicing in short reads. Bioinformatics. 2010;26(7):873-81. Epub 2010/02/12. doi: 10.1093/bioinformatics/btq057. PubMed PMID: 20147302; PubMed Central PMCID: PMC2844994.
281. Mortazavi A, Williams BA, McCue K, Schaeffer L, Wold B. Mapping and quantifying mammalian transcriptomes by RNA-Seq. Nature methods. 2008;5(7):621-8. Epub 2008/06/03. doi: 10.1038/nmeth.1226. PubMed PMID: 18516045.
282. Li B, Ruotti V, Stewart RM, Thomson JA, Dewey CN. RNA-Seq gene expression estimation with read mapping uncertainty. Bioinformatics. 2010;26(4):493-500. Epub 2009/12/22. doi: 10.1093/bioinformatics/btp692. PubMed PMID: 20022975; PubMed Central PMCID: PMC2820677.
283. Sultan M, Amstislavskiy V, Risch T, Schuette M, Dokel S, Ralser M, et al. Influence of RNA extraction methods and library selection schemes on RNA-seq data. BMC genomics. 2014;15:675. Epub 2014/08/13. doi: 10.1186/1471-2164-15-675. PubMed PMID: 25113896; PubMed Central PMCID: PMC4148917.
284. Wan Q, Dingerdissen H, Fan Y, Gulzar N, Pan Y, Wu TJ, et al. BioXpress: an integrated RNA-seq-derived gene expression database for pan-cancer analysis. Database : the journal of biological databases and curation. 2015;2015. Epub 2015/03/31. doi: 10.1093/database/bav019. PubMed PMID: 25819073.
285. Keen JC, Moore HM. The Genotype-Tissue Expression (GTEx) Project: Linking Clinical Data with Molecular Analysis to Advance Personalized Medicine. Journal of personalized medicine. 2015;5(1):22-9. Epub 2015/03/27. doi: 10.3390/jpm5010022. PubMed PMID: 25809799.
286. Kvam VM, Liu P, Si Y. A comparison of statistical methods for detecting differentially expressed genes from RNA-seq data. American journal of botany. 2012;99(2):248-56. Epub 2012/01/24. doi: 10.3732/ajb.1100340. PubMed PMID: 22268221.
287. Risso D, Ngai J, Speed TP, Dudoit S. Normalization of RNA-seq data using factor analysis of control genes or samples. Nature biotechnology. 2014;32(9):896-902. Epub 2014/08/26. doi: 10.1038/nbt.2931. PubMed PMID: 25150836; PubMed Central PMCID: PMC4404308.
